# Expertise and information: an epistemic logic perspective

**DOI:** 10.1007/s11229-023-04064-y

**Published:** 2023-02-13

**Authors:** Joseph Singleton, Richard Booth

**Affiliations:** Cardiff University, Cardiff, UK

**Keywords:** Expertise, Knowledge, Modal logic, Epistemic logic

## Abstract

In this paper we present a modal logic framework to reason about the expertise of information sources. A source is considered an expert on a proposition $\varphi$ if they are able to correctly refute $\varphi$ in any possible world where $\varphi$ is false. Closely connected with expertise is a notion of *soundness of information*: $\varphi$ is said to be “sound” if it is true *up to lack of expertise* of the source. That is, any statement logically weaker than $\varphi$ on which the source has expertise must in fact be true. This is relevant for modelling situations in which sources make claims beyond their domain of expertise. Particular attention is paid to the connection between expertise and *knowledge*: we show that expertise and soundness admit precise interpretations in terms of *S4 and S5 epistemic logic*, under certain conditions. We go on to extend the framework to multiple sources, defining two notions of *collective expertise*. These also have epistemic interpretations via distributed and common knowledge from multi-agent epistemic logic. On the technical side, we give several sound and complete axiomatisations of various classes of expertise models.

## Introduction

In order to properly assess incoming information, it is important to consider the expertise of the reporting source. We should generally believe statements within the domain of expertise of the source, but ignore (or otherwise discount) statements about which the source has no expertise. This applies even when dealing with honest sources: a well-meaning but non-expert source may make false claims due to lack of expertise on the relevant facts. The situation may be further complicated if a source comments on multiple topics at once: we must *filter out* the parts of the statement within their domain of expertise.

Problems associated with expertise have been exacerbated recently by the COVID-19 pandemic, in which false information from non-experts has been shared widely on social media (van Dijck & Alinejad, 2020; Llewellyn, 2020). There have also been high-profile instances of experts going beyond their area of expertise to comment on issues of public health (Xaudiera & Cardenal, 2020), highlighting the importance of *domain-specific* notions of expertise. Identifying experts is also an important task for *liquid democracy* (Blum & Zuber, 2016), in which voters may delegate their votes to expertise on a given policy issue.

Expertise has been well-studied, with perspectives from behavioural and cognitive science (Chi et al., 2014; Ericsson & Towne, 2010), sociology (Collins & Evans, 2008), and philosophy (Goldman, 2018; Kilov, 2021; Whyte & Crease, 2010), among other fields. In this work we study the *logical* content of expertise, and its relation to truthfulness of information.

Specifically, we generalise the *modal logic* setting of Singleton (2021). The two core notions of the framework are *expertise* and *soundness of information*. Intuitively, a source has expertise on $\varphi$ if they are able to correctly refute $\varphi$ in any situation where it is false.^1^ Thus, our notion of expertise *does not depend on the “actual” state of affairs*, but only on the source’s epistemic state.

It is *sound* for a source to report $\varphi$ if $\varphi$ is true *up to lack of expertise*: if $\varphi$ is logically weakened to a proposition $\psi$ on which the source has expertise, then $\psi$ must be true. That is, the consequences of $\varphi$ on which the source has expertise are true. This formalises the idea of “filtering out” parts of a statement within a source’s expertise. For example, suppose $\varphi = p \wedge q$, and the source has expertise on *p* but not *q*. Supposing *p* is true but *q* is false, $\varphi$ is false. However, if we discard information by ignoring *q* (on which the source has no expertise), we obtain the weaker formula *p*, on which the source *does* have expertise, and which is true. If this holds for all possible ways to weaken $p \wedge q$ (this is the case, for instance, if the source does not have expertise on any statement strictly stronger than *p*), then $p \wedge q$ is *false* but *sound* for the source to report.

In terms of refutation, $\varphi$ is sound if the source cannot refute $\lnot \varphi$. That is, either $\varphi$ is in fact true, or the source does not possess sufficient expertise to rule out $\varphi$. This informal picture of expertise already suggests a close connection between expertise, soundness and *knowledge*. Indeed, we will see that, under certain conditions, expertise can be equivalently interpreted in terms of *S4 or S5 knowledge*, familiar from epistemic logic.

Beyond the individual expertise of a single source, one can also consider the *collective expertise* of a group. For example, a committee may consist of several experts across different domains, so that by working together the group achieves expertise beyond any of its individual members. Indeed, such pooling of expertise becomes necessary in cases where it is infeasible for an individual to be a specialist in all relevant sub-areas. As a concrete example, consider the *Rogers Commission report*^2^ into the 1986 Challenger disaster, whose members included politicians, military generals, physicists, astronauts and rocket scientists. Beyond extending the expertise of its constituents, the breadth of expertise among the commission allowed it to collectively assess issues at the *intersection* of its members’ specialities.

Towards defining collective expertise we will again turn to (multi-agent) epistemic logic, borrowing from the well-known notions of *distributed* and *common knowledge* (Fagin et al., 2003). Just as individual expertise (and soundness) can be expressed in terms of knowledge, we will see that collective expertise can be expressed in terms of collective knowledge.

**Contributions.** On the conceptual side, we extend the modal framework of expertise of Singleton (2021) to reason about the expertise of sources and soundness of information. We generalise this framework by working with a more general semantics and introducing collective expertise among multiple sources. On the technical side we obtain axiomatisations for the more general semantics, and axiomatise several new sub-classes of models with additional axioms.

**Paper outline.** In Sect. 2 we give a motivating example and define the syntax and semantics. Section 3 looks at how expertise may be closed under certain operations (e.g. conjunction, negation). The core connection with epistemic logic is given in Sect. 4. We turn to axiomatics in Sect. 5, and give sound and complete logics for various classes of expertise models. In Sect. 6 we generalise to multiple sources, and Sect. 7 concludes. Where proofs are omitted or only sketched, the full details can be found in the appendix.^3^ Several of the main proofs have also been formalised with the Lean theorem prover.^4^

## Expertise and soundness

Before the formal definitions we give an example to illustrate the notions of *expertise* and *soundness*, which are central to the framework.

### Example 1

Consider an economist reporting on the possible impact of a novel virus which has recently been detected. The virus may or may not be highly infectious (*i*) and go on to cause a high death toll (*d*), and there may or may not be economic prosperity in the near future (*p*). The economist reports that despite the virus, the economy will prosper and there will not be mass deaths ($p \wedge \lnot d$). Assume the economist is an expert on matters relating to the economy ($\textsf{E} p$, $\textsf{E} \lnot p$), but not on matters of public health ($\lnot \textsf{E} d$, $\lnot \textsf{E} \lnot d$). For the sake of the example, suppose the virus will in fact cause a high death toll, but the economy will nonetheless prosper. Then while the report of $p \wedge \lnot d$ is false, it is true if one *ignores the parts on which the economist has no expertise* (namely, $\lnot d$); in doing so we obtain *p*, which is true. The report therefore carries *some* true information, even though it is false. We say $p \wedge \lnot d$ is *sound* for the economist in this case.

### Syntax

Let $\textsf{Prop}$ be a countable set of atomic propositions. To start with, we consider a single information source. Our language $\mathcal {L}$ includes modal operators to express expertise and soundness statements for this source, and is defined by the following grammar:

$$\begin{aligned} \varphi {:}{:}{=} p \mid \varphi \wedge \varphi \mid \lnot \varphi \mid \textsf{E} \varphi \mid \textsf{S} \varphi \mid \textsf{A} \varphi \end{aligned}$$

for $p \in \textsf{Prop}$. We read $\textsf{E} \varphi$ as “the source has expertise on $\varphi$, and $\textsf{S} \varphi$ has “$\varphi$ is sound for the source to report”. We include the universal modality $\textsf{A}$ (Goranko & Passy, 1992) for technical convenience; $\textsf{A} \varphi$ is read as “$\varphi$ holds in all states”. Other logical connectives ($\vee$, $\rightarrow$, $\leftrightarrow$) and constants ($\top$, $\bot$) are introduced as abbreviations.

### Semantics

On the semantic side, we use the notion of an *expertise model*.

#### Definition 1

An *expertise model* (hereafter, just *model*) is a triple $M = (X, P, V)$, where *X* is a set of states, $P \subseteq 2^X$ is a collection of subsets of *X*, and $V: \textsf{Prop} \rightarrow 2^X$ is a valuation function. An *expertise frame* is a pair $F = (X, P)$. The class of all models is denoted by $\mathbb {M}$.

The sets in *P* are termed *expertise sets*, and represent the propositions on which the source has expertise. Given the earlier informal description of expertise as refutation, we interpret $A \in P$ as saying that whenever the “actual” state is outside *A*, the source knows so.

For an expertise model $M = (X, P, V)$, the satisfaction relation between states $x \in X$ and formulas $\varphi \in \mathcal {L}$ is defined recursively as follows:

$$\begin{aligned} \begin{array}{lll} M, x &{}\models p &{}\iff x \in V(p) \\ M, x &{}\models \varphi \wedge \psi &{}\iff M, x \models \varphi \text { and } M, x \models \psi \\ M, x &{}\models \lnot \varphi &{}\iff M, x \not \models \varphi \\ M, x &{}\models \textsf{E} \varphi &{}\iff \Vert \varphi \Vert _M \in P \\ M, x &{}\models \textsf{S} \varphi &{}\iff \forall A \in P: \Vert \varphi \Vert _M \subseteq A \implies x \in A \\ M, x &{}\models \textsf{A} \varphi &{}\iff \forall y \in X: M, y \models \varphi \end{array} \end{aligned}$$

where $\Vert \varphi \Vert _M = \{x \in X \mid M, x \models \varphi \}$ is the truth set of $\varphi$. For an expertise frame $F = (X, P)$, write $F \models \varphi$ iff $M, x \models \varphi$ for all models *M* based on *F* and all $x \in X$. Write $M \models \varphi$ iff $M, x \models \varphi$ for all $x \in X$, and $\models \varphi$ iff $M \models \varphi$ for all models *M*; we say $\varphi$ is *valid* in this case. Write $\varphi \equiv \psi$ iff $\varphi \leftrightarrow \psi$ is valid. For a set $\Gamma \subseteq \mathcal {L}$, write $\Gamma \models \varphi$ iff for all models *M* and states *x*, if $M, x \models \psi$ for all $\psi \in \Gamma$ then $M, x \models \varphi$.

The clauses for atomic propositions and propositional connectives are standard. For expertise formulas, we have that $\textsf{E} \varphi$ holds exactly when the set of states where $\varphi$ is true is an element of *P*. Expertise is thus a special case of the *neighbourhood semantics* (Montague, 1970; Pacuit, 2017; Scott, 1970), where each point $x \in X$ has the same set of neighbourhoods. The clause for soundness reflects the intuition that $\varphi$ is sound exactly when all logically weaker formulas on which the source has expertise must be true: if $A \in P$ (i.e. the source has expertise on *A*) and *A* contains all $\varphi$ states, then $x \in A$. In terms of refutation, $\textsf{S} \varphi$ holds iff there is no expertise set *A*, false at the actual state *x*, which allows the source to rule out $\varphi$.

Our truth conditions for expertise and soundness also have topological interpretations, if one views *P* as the collection of closed sets of a topology on *X*^5^$\textsf{E} \varphi$ holds iff $\Vert \varphi \Vert _M$ is closed, and $\textsf{S} \varphi$ holds at *x* iff *x* lies in the *closure* of $\Vert \varphi \Vert _M$.^6^ In this case we can view the closure operation as *expanding* the set $\Vert \varphi \Vert _M$ along the lines of the source’s expertise; $\varphi$ is sound if the “actual” state *x* is included in this expansion. Finally, the clause for the universal modality $\textsf{A}$ states that $\textsf{A} \varphi$ holds iff $\varphi$ holds at all states $y \in X$.

#### Example 2

**Fig. 1:**
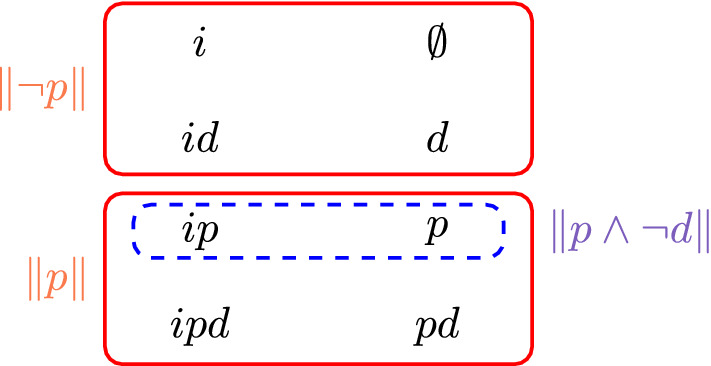
Expertise model from Example 2, which formalises Example 1

To formalise Example 1, consider the model $M = (X, P, V)$ shown in Fig. 1, where $X = 2^{\{i,p,d\}}$, $P = \{\{ipd,pd,ip,p\}, \{id,d,i,\emptyset \}\}$ (indicated by the solid rectangles; sets in *X* are written as strings for brevity), and $V(q) = \{S \mid q \in S\}$. Then we have $M \models \textsf{E} p$ but $M \not \models \textsf{E} d$. The economist’s report of $p \wedge \lnot d$ is represented by the dashed region. We see that while $M, ipd \not \models p \wedge \lnot d$, all expertise sets containing the dashed region also contain *ipd*, so $M, ipd \models \textsf{S} (p \wedge \lnot d)$. That is, the economist’s report is false but sound if the “actual” state of the world were *ipd*. This act of “expanding” $\Vert p \wedge \lnot d\Vert$ until we reach an expertise set corresponds to ignoring the parts of the report on which the economist has no expertise, as in Example 1.

We further illustrate the semantics by listing some valid formulas.

#### Proposition 1

The following formulas are valid:

1. $$\varphi \rightarrow \textsf{S} \varphi $$
2. $$\textsf{E} \varphi \leftrightarrow \textsf{A} \textsf{E} \varphi $$
3. $$\textsf{A} (\varphi \rightarrow \psi ) \rightarrow (\textsf{S} \varphi \wedge \textsf{E} \psi \rightarrow \psi )$$
4. $$\textsf{E} \varphi \rightarrow \textsf{A} (\textsf{S} \varphi \rightarrow \varphi )$$

#### Proof

Let $M = (X, P, V)$ be a model and $x \in X$. (1) and (2) are clear. For (3), suppose $M, x \models \textsf{A} (\varphi \rightarrow \psi )$. Then $\Vert \varphi \Vert _M \subseteq \Vert \psi \Vert _M$. Further, suppose $M, x \models \textsf{S} \varphi \wedge \textsf{E} \psi$. Then $\Vert \varphi \Vert _M \subseteq \Vert \psi \Vert _M \in P$; taking $A = \Vert \psi \Vert _M$ in the definition of the semantics for $\textsf{S}$, we get by $M, x \models \textsf{S} \varphi$ that $x \in \Vert \psi \Vert _M$, i.e. $M, x \models \psi$. Finally, (4) follows from (2) and (3) by taking $\psi = \varphi$. $\square$

Here (1) says that all truths are sound. (2) says that expertise is global. (3) says that if the source has expertise on $\psi$, and $\psi$ is logically weaker than some sound formula $\varphi$, then $\psi$ is in fact true. This formalises the idea that if $\varphi$ is true *up to lack of expertise*, then weakening $\varphi$ until expertise holds (i.e. discarding parts of $\varphi$ on which the source does not have expertise) results in something true. (4) says that if the source has expertise on $\varphi$, then whenever $\varphi$ is sound it is also true.

## Closure properties

So far we have not imposed any constraints on the collection of expertise sets *P*. But given our interpretation of *P*, it may be natural to require that *P* is closed under certain set-theoretic operations. Say a frame $F = (X, P)$ is

- *closed under intersections* if $\{A_i\}_{i \in I} \subseteq P$ implies $\bigcap _{i \in I}{A_i} \in P$
- *closed under unions* if $\{A_i\}_{i \in I} \subseteq P$ implies $\bigcup _{i \in I}{A_i} \in P$
- *closed under finite unions* if $A, B \in P$ implies $A \cup B \in P$
- *closed under complements* if $A \in P$ implies $X {\setminus } A \in P$

In the first two cases we allow the empty collection $\emptyset \subseteq P$, and employ the nullary intersection convention $\bigcap \emptyset = X$. Consequently, closure under intersections implies $X \in P$, and closure under unions implies $\emptyset \in P$.

Say a model has any of the above properties if the underlying frame does. Write $\mathbb {M}_{\textsf{int} }$, $\mathbb {M}_{\textsf{unions} }$, $\mathbb {M}_{\textsf{unions} }$, $\mathbb {M}_{\mathsf {finite-unions} }$ and $\mathbb {M}_{\textsf{compl} }$ for the classes of models closed under intersections, unions, finite unions and complements respectively.

What are the intuitive interpretations of these closure conditions? Consider again our interpretation of $A \in P$: whenever the actual state is not in *A*, the source knows so. With this in mind, closure under intersections is a natural property: if $x \notin \bigcap _{i \in I}{A_i}$ then there is some $i \in I$ such that $x \notin A_i$; the source can then use this to refute $A_i$ and therefore know that the actual state *x* does not lie in the intersection $\bigcap _{i \in I}{A_i}$. A similar argument can be made for finite unions: if $x \notin A \cup B$ then the source can use $x \notin A$ and $x \notin B$ to refute both *A* and *B*. Closure under *arbitrary* unions is less clear cut; determining that $x \notin \bigcup _{i \in I}{A_i}$ requires the source to refute (potentially) infinitely many propositions $A_i$. This is more demanding from a computational and cognitive perspective, and we therefore view closure under (arbitrary) unions as an optional property which may or may not be appropriate depending on the situation one wishes to model. Finally, closure under complements removes the distinction between refutation and *verification*: if the agent can refute *A* whenever *A* is false, they can also verify *A* whenever *A* is true. We view this as another optional property, which is appropriate in situations where *symmetric* expertise is desirable (i.e. when expertise on $\varphi$ and $\lnot \varphi$ should be considered equivalent).

Several of these properties can be formally captured in our language at the level of frames.

### Proposition 2

Let $F = (X, P)$ be a non-empty frame. Then

1. *F* is closed under intersections iff $F \models \textsf{A} (\textsf{S} \varphi \rightarrow \varphi ) \rightarrow \textsf{E} \varphi$ for all $\varphi \in \mathcal {L}$
2. *F* is closed under finite unions iff $F \models \textsf{E} \varphi \wedge \textsf{E} \psi \rightarrow \textsf{E} (\varphi \vee \psi )$ for all $\varphi \in \mathcal {L}$
3. *F* is closed under complements iff $F \models \textsf{E} \varphi \leftrightarrow \textsf{E} \lnot \varphi$ for all $\varphi \in \mathcal {L}$

### Proof

We prove only the first claim; the others are straightforward.

“if”: We show the contrapositive. Suppose *F* is not closed under intersections. Then there is a collection $\{A_i\}_{i \in I} \subseteq P$ such that $B {:}{=} \bigcap _{i \in I}A_i \notin P$. Let *p* be an arbitrary atomic proposition, and define a valuation *V* by $V(p) = B$ and $V(q) = \emptyset$ for $q \ne p$. Let $M = (X, P, V)$ be the corresponding model. Since *X* is assumed to be non-empty, we may take some $x \in X$.

We claim that $M, x \models \textsf{A} (\textsf{S} p \rightarrow p)$ but $M, x \not \models \textsf{E} p$. Clearly $M, x \not \models \textsf{E} p$ since $\Vert p\Vert _M = B \notin P$. For $M, x \models \textsf{A} (\textsf{S} p \rightarrow p)$, suppose $y \in X$ and $M, y \models \textsf{S} p$. Let $j \in I$. Then $A_j \in P$, and

$$\begin{aligned} \Vert p\Vert _M = B = \bigcap _{i \in I}{A_i} \subseteq A_j \end{aligned}$$

so by $M, y \models \textsf{S} p$ we have $y \in A_j$. Hence $y \in \bigcap _{j \in I}A_j = B = \Vert p\Vert _M$, so $M, y \models p$. This shows that any $y \in X$ has $M, y \models \textsf{S} p \rightarrow p$, and thus $M, x \models \textsf{A} (\textsf{S} p \rightarrow p)$. Hence $F \not \models \textsf{A} (\textsf{S} p \rightarrow p) \rightarrow \textsf{E} p$.

“only if”: Suppose *F* is closed under intersections. Let *M* be a model based on *F* and take $x \in X$. Let $\varphi \in \mathcal {L}$. Suppose $M, x \models \textsf{A} (\textsf{S} \varphi \rightarrow \varphi )$. Then $\Vert \textsf{S} \varphi \Vert _M \subseteq \Vert \varphi \Vert _M$. But since $\models \varphi \rightarrow \textsf{S} \varphi$, we have $\Vert \varphi \Vert _M \subseteq \Vert \textsf{S} \varphi \Vert _M$ too. Hence $\Vert \varphi \Vert _M = \Vert \textsf{S} \varphi \Vert _M$, i.e.

$$\begin{aligned} \Vert \varphi \Vert _M = \Vert \textsf{S} \varphi \Vert _M = \bigcap \{A \in P \mid \Vert \varphi \Vert _M \subseteq A\} \in P \end{aligned}$$

where we use the fact that *P* is closed under intersections in the final step. Hence $\Vert \varphi \Vert _M \in P$, so $M, x \models \textsf{E} \varphi$. $\square$

The question of whether closure under (arbitrary) unions can be expressed in the language is still open. By Propositions 2 (1) and 1 (4), the language fragment $\mathcal {L}_{\textsf{S} \textsf{A} }$ containing only the $\textsf{S}$ and $\textsf{A}$ modalities is equally expressive as the full language $\mathcal {L}$ with respect to $\mathbb {M}_{\textsf{int} }$, since $\textsf{E} \varphi$ is equivalent to $\textsf{A} (\textsf{S} \varphi \rightarrow \varphi )$ in such models. In general $\mathcal {L}_{\textsf{S} \textsf{A} }$ is strictly less expressive, since $\mathcal {L}_{\textsf{S} \textsf{A} }$ cannot distinguish between a model and its closure under intersections.

### Lemma 1

Let $M = (X, P, V)$ be a model, and $M' = (X, P', V)$ its closure under intersections, where $A \in P'$ iff $A = \bigcap _{i \in I}{A_i}$ for some $\{A_i\}_{i \in I} \subseteq P$. Then for all $\varphi \in \mathcal {L}_{\textsf{S} \textsf{A} }$ and $x \in X$, we have $M, x \models \varphi$ iff $M', x \models \varphi$.

### Proof

By induction on $\mathcal {L}_{\textsf{S} \textsf{A} }$ formulas. The cases for atomic propositions, propositional connectives and $\textsf{A}$ are straightforward. We treat only the case for $\textsf{S}$.

The “if” direction is clear using the induction hypothesis and the fact that $P \subseteq P'$. Suppose $M, x \models \textsf{S} \varphi$. Take $A = \bigcap _{i \in I}{A_i} \in P'$, where each $A_i$ is in *P*, such that $\Vert \varphi \Vert _{M'} \subseteq A$. By the induction hypothesis, $\Vert \varphi \Vert _M \subseteq A$. For any $i \in I$, $\Vert \varphi \Vert _M \subseteq A \subseteq A_i$ and $M, x \models \textsf{S} \varphi$ gives $x \in A_i$. Hence $x \in \bigcap _{i \in I}{A_i} = A$. This shows $M', x \models \textsf{S} \varphi$. $\square$

It follows that $\mathcal {L}_{\textsf{S} \textsf{A} }$ is strictly less expressive than $\mathcal {L}$.^7^ To round off the discussion of closure properties, we note that within the class of frames closed under intersections, closure under finite unions is also captured by the well-known **K** axiom—$\Box (\varphi \rightarrow \psi ) \rightarrow (\Box \varphi \rightarrow \Box \psi )$—for the dual soundness operator $\hat{\textsf{S} }\varphi \,{:}{=}\, \lnot \textsf{S} \lnot \varphi$:

### Proposition 3

Suppose $F = (X, P)$ is non-empty and closed under intersections. Then *F* is closed under finite unions if and only if $F \models \hat{\textsf{S} }(\varphi \rightarrow \psi ) \rightarrow (\hat{\textsf{S} }\varphi \rightarrow \hat{\textsf{S} }\psi )$ for all $\varphi , \psi \in \mathcal {L}$.

### Proof

“if”: We show the contrapositive. Suppose *F* is closed under intersections but not finite unions, so that there are $B_1, B_2 \in P$ with $B_1 \cup B_2 \notin P$. Set

$$\begin{aligned} C = \bigcap \{A \in P \mid B_1 \cup B_2 \subseteq A\} \end{aligned}$$

By closure under intersections, $C \in P$. Clearly $B_1 \cup B_2 \subseteq C$. Since $C \in P$ but $B_1 \cup B_2 \notin P$, $B_1 \cup B_2 \subset C$. Hence there is $x \in C {\setminus } (B_1 \cup B_2)$.

Now pick distinct atomic propositions *p* and *q*, and let *V* be any valuation with $V(p) = B_1 \cup B_2$ and $V(q) = B_1$. Let $M = (X, P, V)$ be the corresponding model. We make three claims:

- $M, x \models \textsf{S} p$: Take $A \in P$ such that $\Vert p\Vert _M \subseteq A$. Then $B_1 \cup B_2 \subseteq A$, so $C \subseteq A$. Since $x \in C$, we have $x \in A$ as required.
- $M, x \not \models \textsf{S} q$: This is clear since $B_1 \in P$, $\Vert q\Vert _M \subseteq B_1$, but $x \notin B_1$.
- $M, x \not \models \textsf{S} (p \wedge \lnot q)$: Note that $\Vert p \wedge \lnot q\Vert _M = V(p) {\setminus } V(q) = B_2 {\setminus } B_1$. Therefore we have $B_2 \in P$ and $\Vert p \wedge \lnot q\Vert _M \subseteq B_2$, but $x \notin B_2$.

Now set $\varphi = \lnot q$ and $\psi = \lnot p$. We have

$$\begin{aligned} \hat{\textsf{S} }(\varphi \rightarrow \psi )= & {} \lnot \textsf{S} \lnot (\varphi \rightarrow \psi ) \equiv \lnot \textsf{S} (\varphi \wedge \lnot \psi ) \equiv \lnot \textsf{S} (p \wedge \lnot q)\\ \hat{\textsf{S} }\varphi \rightarrow \hat{\textsf{S} }\psi= & {} \lnot \textsf{S} \lnot \varphi \rightarrow \lnot \textsf{S} \lnot \psi \equiv \lnot \textsf{S} q \rightarrow \lnot \textsf{S} p \equiv \textsf{S} p \rightarrow \textsf{S} q \end{aligned}$$

From the claims above we see that $M, x \models \hat{\textsf{S} }(\varphi \rightarrow \psi )$ but $M, x \not \models \hat{\textsf{S} }\varphi \rightarrow \hat{\textsf{S} }\psi$. Since *M* is a model based on *F*, we are done.

“only if”: Suppose *F* is closed under intersections and finite unions. Let *M* be a model based on *F* and *x* a state in *M*. Suppose $M, x \models \hat{\textsf{S} }(\varphi \rightarrow \psi )$ and $M, x \models \hat{\textsf{S} }\varphi$. Then $M, x \not \models \textsf{S} \lnot (\varphi \rightarrow \psi )$ and $M, x \not \models \textsf{S} \lnot \varphi$. Hence there is $A \in P$ such that $\Vert \lnot (\varphi \rightarrow \psi )\Vert _M \subseteq A$ but $x \notin A$, and $B \in P$ such that $\Vert \lnot \varphi \Vert _M \subseteq B$ but $x \notin B$. Note

$$\begin{aligned} \Vert \lnot \psi \Vert _M \subseteq \Vert \varphi \wedge \lnot \psi \Vert _M \cup \Vert \lnot \varphi \Vert _M = \Vert \lnot (\varphi \rightarrow \psi )\Vert _M \cup \Vert \lnot \varphi \Vert _M \subseteq A \cup B. \end{aligned}$$

Since $x \notin A \cup B$ and $A \cup B \in P$ by closure under finite unions, this shows $M, x \not \models \textsf{S} \lnot \psi$, i.e. $M, x \models \hat{\textsf{S} }\psi$. This completes the proof of $F \models \hat{\textsf{S} }(\varphi \rightarrow \psi ) \rightarrow (\hat{\textsf{S} }\varphi \rightarrow \hat{\textsf{S} }\psi )$. $\square$

## Connection with epistemic logic

In this section we explore the connection between our logic and *epistemic logic*, for certain classes of expertise models. In particular, we show a one-to-one mapping between classes of expertise models and *S4 and S5 relational models*, and a translation from $\mathcal {L}$ to the modal language with knowledge operator $\textsf{K}$ which allows expertise and soundness to be expressed in terms of *knowledge*.

First, we introduce the syntax and (relational) semantics of epistemic logic. Let $\mathcal {L}_{\textsf{K} \textsf{A} }$ be the language formed from $\textsf{Prop}$ with modal operators $\textsf{K}$ and $\textsf{A}$. We read $\textsf{K} \varphi$ as *the source knows* $\varphi$.

### Definition 2

A *relational model* is a triple $M^* = (X, R, V)$, where *X* is a set of states, $R \subseteq X \times X$ is a binary relation on *X*, and $V: \textsf{Prop} \rightarrow 2^X$ is a valuation function. The class of all relational models is denoted by $\mathbb {M}^*$.

The satisfaction relation for $\mathcal {L}_{\textsf{K} \textsf{A} }$ is defined recursively: the clauses for atomic propositions, propositional connectives and $\textsf{A}$ are the same as for expertise models, and

$$\begin{aligned} M^*, x \models \textsf{K} \varphi \iff \forall y \in X: xRy \implies M^*, y \models \varphi . \end{aligned}$$

As is standard, *R* is interpreted as an *epistemic accessibility relation*: *xRy* means that the source considers *y* possible if the “actual” state of the world is *x*. We will be interested in the logics of S4 and S5, which are axiomatised by **KT4** and **KT5**, respectively:

- **K**: $$\textsf{K} (\varphi \rightarrow \psi ) \rightarrow (\textsf{K} \varphi \rightarrow \textsf{K} \psi )$$
- **T**: $$\textsf{K} \varphi \rightarrow \varphi $$
- **4**: $$\textsf{K} \varphi \rightarrow \textsf{K} \textsf{K} \varphi $$
- **5**: $$\lnot \textsf{K} \varphi \rightarrow \textsf{K} \lnot \textsf{K} \varphi $$

**T** says that all knowledge is true, **4** expresses *positive introspection* of knowledge, and **5** expresses *negative introspection*.

It is well known that S4 is sound and complete for the class of relational models where *R* is reflexive and transitive, and that S5 is sound and complete for the class of relational models where *R* is an equivalence relation. Accordingly, we write $\mathbb {M}^*_{\textsf{S4} }$ for the class of all $M^*$ where *R* is reflexive and transitive, and $\mathbb {M}^*_{\textsf{S5} }$ for $M^*$ where *R* is an equivalence relation.

Our first result connecting expertise and knowledge is on the semantic side: we show there is a bijection between expertise models closed under intersections and unions and S4 models. Moreover, there is a close connection between the collection of expertise sets *P* and the corresponding relation *R*. Since expertise models closed under intersections and unions are *Alexandrov topological spaces* (where *P* is the set of closed sets), this is essentially a reformulation of a known result linking relational semantics over S4 frames and topological interior semantics over Alexandrov spaces (Özgün, 2017; van Benthem & Bezhanishvili, 2007).^8^ To be self-contained, we prove it for our setting here. First, we show how to map a collection of sets *P* to a binary relation.

### Definition 3

For a set *X* and $P \subseteq 2^X$, let $R_P$ be the binary relation on *X* given by

$$\begin{aligned} x{R_P}y \iff \forall A \in P: (y \in A \implies x \in A) \end{aligned}$$

**Fig. 2:**
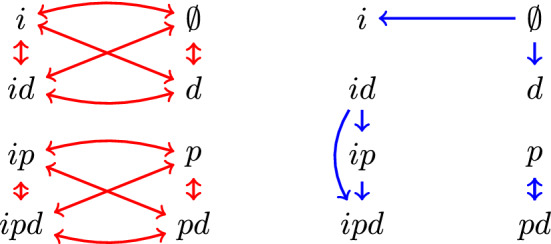
Left: the relation $R_P$ corresponding to *X* and *P* from Example 2 (with reflexive edges omitted). Note that $R_P$ is an equivalence relation, with equivalence classes $\Vert p\Vert$ and $\Vert \lnot p\Vert$. Right: an example of a non-symmetric relation $R_P$, corresponding to $P = \{ \emptyset , X, \{id,ip,ipd\}, \{id,ip\}, \{id\}, \{i,\emptyset \}, \{\emptyset ,d\}, \{p,pd\} \}$

In the case where *P* is the collection of closed sets of a topology on *X*, $R_P$ is the *specialisation preorder*. Figure 2 shows an example of $R_P$ for *X* and *P* from Example 2. In what follows, say a set $A \subseteq X$ is *downwards closed* with respect to a relation *R* if *xRy* and $y \in A$ implies $x \in A$.

### Lemma 2

Let *X* be a set and *R*, *S* reflexive and transitive relations on *X*. Then if *R* and *S* share the same downwards closed sets, $R = S$.

### Proof

Suppose *xRy*. Set $A = \{z \in X \mid zSy\}$. By transitivity of *S*, *A* is downwards closed wrt *S*. By assumption, *A* must also be downwards closed wrt *R*. By reflexivity of *S*, $y \in A$. Hence *xRy* implies $x \in A$, i.e. *xSy*. This shows $R \subseteq S$, and the reverse inclusion holds by a symmetrical argument. Hence $R = S$. $\square$

### Lemma 3

Let *X* be a set.

1. For any $P \subseteq 2^X$, $R_P$ is reflexive and transitive.
2. If $P \subseteq 2^X$ is closed under unions and intersections, then for all $A \subseteq X$:
  $$\begin{aligned} A \in P \iff A \text { is downwards closed wrt } R_P. \end{aligned}$$
3. If *R* is a reflexive and transitive relation on *X*, there is $P \subseteq 2^X$ closed under unions and intersections such that $R_P = R$.

### Proof

1. Straightforward by the definition of $R_P$.
2. Suppose *P* is closed under unions and intersections and let $A \subseteq X$. First suppose $A \in P$. Then *A* is downwards closed with respect to $R_P$: if $y \in A$ and $x{R_P}y$ then, by definition of $R_P$, we have $x \in A$.
  Next suppose *A* is downwards closed with respect to $R_P$. We claim
  $$\begin{aligned} A = \bigcup _{y \in A}\bigcap \{B \in P \mid y \in B\} \end{aligned}$$
  Since *P* is closed under intersections and unions, this will show $A \in P$. The left-to-right inclusion is clear, since any $y \in A$ lies in the term of the union corresponding to *y*. For the right-to-left inclusion, take any *x* in the set on the RHS. Then there is $y \in A$ such that $x \in \bigcap \{B \in P \mid y \in B\}$. But this is just a rephrasing of $x{R_P}y$. Since *A* is downwards closed, we get $x \in A$ as required.
3. Take any reflexive and transitive relation *R*. Set
  $$\begin{aligned} P = \{A \subseteq X \mid A \text { is downwards closed wrt } R\}. \end{aligned}$$
  It is easily seen that *P* is closed under unions and intersections. We need to show that $R_P = R$. By (1), $R_P$ is reflexive and transitive. By Lemma 2, it is sufficient to show that $R_P$ and *R* share the same downwards closed sets. Indeed, for any $A \subseteq X$ we get by (2) and the definition of *P* that
  $$\begin{aligned} \begin{aligned} A \text { is downwards closed wrt } R_P&\iff A \in P \\&\iff A \text { is downwards closed wrt } R. \end{aligned} \end{aligned}$$

$$\square $$

We can now state the correspondence between expertise models and S4 relational models.

### Theorem 1

The mapping $f: \mathbb {M}_{\textsf{int} }\cap \mathbb {M}_{\textsf{unions} }\rightarrow \mathbb {M}^*_{\textsf{S4} }$ given by $(X, P, V) \mapsto (X, R_P, V)$ is bijective.

### Proof

Lemma 3 (1) shows that *f* is well-defined, i.e. that *f*(*M*) does indeed lie in $\mathbb {M}^*_{\textsf{S4} }$ for any expertise model *M*. Injectivity follows from Lemma 3 (2), since *P* is fully determined by $R_P$ for expertise models closed under unions and intersections. Finally, Lemma 3 (3) shows that *f* is surjective. $\square$

If we consider closure under complements together with intersections, an analogous result holds with S5 taking the place of S4.

### Theorem 2

The mapping $g: \mathbb {M}_{\textsf{int} }\cap \mathbb {M}_{\textsf{compl} }\rightarrow \mathbb {M}^*_{\textsf{S5} }$ given by $(X, P, V) \mapsto (X, R_P, V)$ is bijective.

### Proof

First, note that $\mathbb {M}_{\textsf{int} }\cap \mathbb {M}_{\textsf{compl} }\subseteq \mathbb {M}_{\textsf{int} }\cap \mathbb {M}_{\textsf{unions} }$, since any union of sets in *P* can be written as a complement of intersection of complements of sets in *P*. Therefore *g* is simply the restriction of *f* from Theorem 1 to $\mathbb {M}_{\textsf{int} }\cap \mathbb {M}_{\textsf{compl} }$.

To show *g* is well-defined, we need to show that $R_P$ is an equivalence relation whenever *P* is closed under intersections and complements. Reflexivity and transitivity were already shown in Lemma 3 (1). We show $R_P$ is symmetric. Suppose $x{R_P}y$. Let $A \in P$ such that $x \in A$. Write $B = X {\setminus } A$. Then since *P* is closed under complements, $B \in P$. Since $x{R_P}y$ and $x \notin B$, we cannot have $y \in B$. Thus $y \notin B = X {\setminus } A$, i.e. $y \in A$. This shows $y{R_P}x$. Hence $R_P$ is an equivalence relation.

Injectivity of *g* is inherited from injectivity of *f* from Theorem 1. For surjectivity, it suffices to show that $f^{-1}(M^*)$ is closed under complements when $M^* = (X, R, V) \in \mathbb {M}^*_{\textsf{S5} }$. Recall, from Lemma 3 (3), that $f^{-1}(M^*) = (X, P, V)$, where $A \in P$ iff *A* is downwards closed with respect to *R*. Suppose $A \in P$, i.e. *A* is downwards closed. To show $X \setminus A$ is downwards closed, suppose $y \in X {\setminus } A$ and *xRy*. By symmetry of *R*, *yRx*. If $x \in A$, then downwards closure of *A* would give $y \in A$, but this is false. Hence $x \notin A$, i.e. $x \in X \setminus A$. Thus $X {\setminus } A$ is downwards closed, so *P* is closed under complements. This completes the proof. $\square$

The mappings between expertise models and relational models also preserve the truth value of formulas, via the following translation $t: \mathcal {L}\rightarrow \mathcal {L}_{\textsf{K} \textsf{A} }$, which expresses expertise and soundness in terms of knowledge:

$$\begin{aligned} t(p)&= p \\ t(\varphi \wedge \psi )&= t(\varphi ) \wedge t(\psi ) \\ t(\lnot \varphi )&= \lnot t(\varphi ) \\ t(\textsf{E} \varphi )&= \textsf{A} (\lnot t(\varphi ) \rightarrow \textsf{K} \lnot t(\varphi )) \\ t(\textsf{S} \varphi )&= \lnot \textsf{K} \lnot t(\varphi ) \\ t(\textsf{A} \varphi )&= \textsf{A} t(\varphi ). \end{aligned}$$

The only interesting cases are for $\textsf{E} \varphi$ and $\textsf{S} \varphi$. The translation of $\textsf{E} \varphi$ corresponds directly to the intuition of expertise as refutation: in all possible scenarios, if $\varphi$ is false the source knows so. The translation of $\textsf{S} \varphi$ says that soundness is just the dual of knowledge: $\varphi$ is sound if the source does not *know* that $\varphi$ is false.

### Theorem 3

Let $f: \mathbb {M}_{\textsf{int} }\cap \mathbb {M}_{\textsf{unions} }\rightarrow \mathbb {M}^*_{\textsf{S4} }$ be the bijection from Theorem 1. Then for all $M = (X, P, V) \in \mathbb {M}_{\textsf{int} }\cap \mathbb {M}_{\textsf{unions} }$, $x \in X$ and $\varphi \in \mathcal {L}$:

1 $$\begin{aligned} M, x \models \varphi \iff f(M), x \models t(\varphi ) \end{aligned}$$

Moreover, if $g: \mathbb {M}_{\textsf{int} }\cap \mathbb {M}_{\textsf{compl} }\rightarrow \mathbb {M}^*_{\textsf{S5} }$ is the bijection from Theorem 2, then for all $M = (X, P, V) \in \mathbb {M}_{\textsf{int} }\cap \mathbb {M}_{\textsf{compl} }$:

2 $$\begin{aligned} M, x \models \varphi \iff g(M), x \models t(\varphi ) \end{aligned}$$

### Proof

Note that since *g* is defined as the restriction of *f* to $\mathbb {M}_{\textsf{int} }\cap \mathbb {M}_{\textsf{compl} }$, (2) follows from (1). We show (1) only. Let $M = (X, P, V) \in \mathbb {M}_{\textsf{int} }\cap \mathbb {M}_{\textsf{unions} }$. Write $f(M) = (X, R, V)$. From the definition of *f* and Lemma 3 (2), we have

$$\begin{aligned} A \in P \iff A \text { is downwards closed wrt R} \qquad (*) \end{aligned}$$

We show (1) by induction. The only non-trivial cases are $\textsf{E}$ and $\textsf{S}$ formulas.

($\textsf{E}$): Suppose $M, x \models \textsf{E} \varphi$. Then $\Vert \varphi \Vert _M \in P$. By the induction hypothesis and $(*)$, this means $\Vert t(\varphi )\Vert _{f(M)}$ is downwards closed with respect to *R*. Now take $y \in X$ such that $f(M), y \models \lnot t(\varphi )$. Then $y \notin \Vert t(\varphi )\Vert _{f(M)}$. Since this set is downwards closed, it cannot contain any *R*-successor of *y*. Hence $f(M), y \models \textsf{K} \lnot t(\varphi )$. This shows that $f(M), x \models \textsf{A} (\lnot t(\varphi ) \rightarrow \textsf{K} \lnot t(\varphi ))$, i.e. $f(M), x \models t(\textsf{E} \varphi )$.

Now suppose $f(M), x \models t(\textsf{E} \varphi )$, i.e. $f(M), x \models \textsf{A} (\lnot t(\varphi ) \rightarrow \textsf{K} \lnot t(\varphi ))$. We show $\Vert \varphi \Vert _M$ is downwards closed. Suppose *yRz* and $z \in \Vert \varphi \Vert _M$. By the induction hypothesis, $f(M), z \not \models \lnot t(\varphi )$. Hence $f(M), y \not \models \textsf{K} \lnot t(\varphi )$. Since $\lnot t(\varphi ) \rightarrow \textsf{K} \lnot t(\varphi )$ holds everywhere in *f*(*M*), this means $f(M), y \models t(\varphi )$; by the induction hypothesis again we get $M, y \models \varphi$ and thus $y \in \Vert \varphi \Vert _M$. This shows that $\Vert \varphi \Vert _M$ is downwards closed, and by $(*)$ we have $\Vert \varphi \Vert _M \in P$. Hence $M, x \models \textsf{E} \varphi$.

($\textsf{S}$): We show both directions by contraposition. Suppose $M, x \not \models \textsf{S} \varphi$. Then there is $A \in P$ such that $\Vert \varphi \Vert _M \subseteq A$ and $x \notin A$. Since *A* is downwards closed (by $(*)$), this means *xRy* implies $y \notin A$ and hence $y \notin \Vert \varphi \Vert _M$, for any $y \in X$. By the induction hypothesis, we get that *xRy* implies $f(M), y \models \lnot t(\varphi )$, i.e. $f(M), x \models \textsf{K} \lnot t(\varphi )$. Hence $f(M), x \not \models t(\textsf{S} \varphi )$.

Finally, suppose $f(M), x \not \models t(\textsf{S} \varphi )$, i.e. $f(M), x \models \textsf{K} \lnot t(\varphi )$. Let *A* be the *R*-downwards closure of $\Vert \varphi \Vert _M$, i.e.

$$\begin{aligned} A = \{y \in X \mid \exists z \in \Vert \varphi \Vert _M: yRz\} \end{aligned}$$

Then $\Vert \varphi \Vert _M \subseteq A$ by reflexivity of *R*, and *A* is downwards closed by transitivity. Hence $A \in P$. But $x \notin A$, since for all *z* with *xRz* we have $f(M), z \models \lnot t(\varphi )$, so $z \notin \Vert t(\varphi )\Vert _{f(M)} = \Vert \varphi \Vert _M$. Hence $M, x \not \models \textsf{S} \varphi$. $\square$

Taken together, the results of this section show that, when considering expertise models closed under intersections and unions, *P* *uniquely determines* an epistemic accessibility relation such that expertise and soundness have precise interpretations in terms of S4 knowledge. If we also impose closure under complements, the notion of knowledge is strengthened to S5. Moreover, every S4 and S5 model arises from some expertise model in this way.

## Axiomatisation

In this section we give sound and complete logics with respect to various classes of expertise models. We start with the class of all expertise models $\mathbb {M}$, and show how adding more axioms captures the closure conditions of Sect. 3.

### The general case

Let $\textsf{L}$ be the extension of propositional logic generated by the axioms and inference rules shown in Table 1. Note that we treat $\textsf{A}$ as a “box” and $\textsf{S}$ as a “diamond” modality. Some of the axioms were already seen in Proposition 1; new ones include “replacement of equivalents” for expertise $(\text{ RE}_\textsf{E} )$, **4** for $\textsf{S}$ $(\text{4 }_{\textsf{S} })$, and $(\text{ W}_{\textsf{S} })$, which says that if $\psi$ is logically weaker than $\varphi$ then the same holds for $\textsf{S} \psi$ and $\textsf{S} \varphi$. First, $\textsf{L}$ is sound.

**Table 1:** Axioms and inference rules for $\textsf{L}$

$\textsf{E} \varphi \leftrightarrow \textsf{A} \textsf{E} \varphi$	$(\text{ EA})$
$\textsf{A} (\varphi \leftrightarrow \psi ) \rightarrow (\textsf{E} \varphi \leftrightarrow \textsf{E} \psi )$	$(\text{ RE}_\textsf{E} )$
$\textsf{A} (\varphi \rightarrow \psi ) \rightarrow (\textsf{S} \varphi \wedge \textsf{E} \psi \rightarrow \psi )$	$(\text{ W}_{\textsf{E} })$
$\varphi \rightarrow \textsf{S} \varphi$	$(\text{ T}_{\textsf{S} })$
$\textsf{S} \textsf{S} \varphi \rightarrow \textsf{S} \varphi$	$(\text{4 }_{\textsf{S} })$
$\textsf{A} (\varphi \rightarrow \psi ) \rightarrow (\textsf{S} \varphi \rightarrow \textsf{S} \psi )$	$(\text{ W}_{\textsf{S} })$
$\textsf{A} (\varphi \rightarrow \psi ) \rightarrow (\textsf{A} \varphi \rightarrow \textsf{A} \psi )$	$(\text{ K}_{\textsf{A} })$
$\textsf{A} \varphi \rightarrow \varphi$	$(\text{ T}_{\textsf{A} })$
$\lnot \textsf{A} \varphi \rightarrow \textsf{A} \lnot \textsf{A} \varphi$	$(\text{5 }_{\textsf{A} })$
From $\varphi$ infer $\textsf{A} \varphi$	$(\text{ Nec}_{\textsf{A} })$
From $\varphi \rightarrow \psi$ and $\varphi$ infer $\psi$	$(\text{ MP})$

#### Lemma 4

$\textsf{L}$ is sound with respect to $\mathbb {M}$.

#### Proof

The inference rules are clearly sound. All axioms were either shown to be sound in Proposition 1 or are straightforward to see, with the possible exception of $(\text{4 }_{\textsf{S} })$ which we will show explicitly. Let $M = (X, P, V)$ be an expertise model and $x \in X$. Suppose $M, x \models \textsf{S} \textsf{S} \varphi$. We need to show $M, x \models \textsf{S} \varphi$. Take $A \in P$ such that $\Vert \varphi \Vert _M \subseteq A$. Now for any $y \in X$, if $M, y \models \textsf{S} \varphi$ then clearly $y \in A$. Hence $\Vert \textsf{S} \varphi \Vert _M \subseteq A$. But then $M, x \models \textsf{S} \textsf{S} \varphi$ gives $x \in A$. Hence $M, x \models \textsf{S} \varphi$. $\square$

For completeness, we use a variation of the standard canonical model method. In taking this approach, one constructs a model whose states are maximally $\textsf{L}$-consistent sets of formulas, and aims to prove the *truth lemma*: that a set $\Gamma$ satisfies $\varphi$ in the canonical model if and only if $\varphi \in \Gamma$. However, the truth lemma poses some difficulties for our semantics. Roughly speaking, we find there is an obvious choice of *P* to ensure the truth lemma for $\textsf{E} \varphi$ formulas, but that this may be too small for $\textsf{S} \varphi$ to be refuted when $\textsf{S} \varphi \notin \Gamma$ (recall that $M, x \not \models \textsf{S} \varphi$ iff *there exists* some $A \in P$ such that $\Vert \varphi \Vert _M \subseteq A$ and $x \notin A$). We therefore “enlargen” the set of states so we can add new expertise sets *A*—without affecting the truth value of expertise formulas—to obtain the truth lemma for soundness formulas.

First, some standard notation and terminology. Write $\vdash \varphi$ iff $\varphi \in \textsf{L}$. For $\Gamma \subseteq \mathcal {L}$ and $\varphi \in \mathcal {L}$, write $\Gamma \vdash \varphi$ iff there are $\psi _0, \ldots , \psi _n \in \Gamma$, $n \ge 0$, such that $\vdash (\psi _0 \wedge \cdots \wedge \psi _n) \rightarrow \varphi$. Say $\Gamma$ is *inconsistent* if $\Gamma \vdash \bot$, and *consistent* otherwise. $\Gamma$ is *maximally consistent* iff $\Gamma$ is consistent and $\Gamma \subset \Delta$ implies that $\Delta$ is inconsistent. We recall some standard facts about maximally consistent sets.

#### Lemma 5

Let $\Gamma$ be a maximally consistent set and $\varphi , \psi \in \mathcal {L}$. Then

1. $$\varphi \in \Gamma $$ iff $$\Gamma \vdash \varphi $$
2. If $\varphi \rightarrow \psi \in \Gamma$ and $\varphi \in \Gamma$, then $\psi \in \Gamma$
3. $$\lnot \varphi \in \Gamma $$ iff $$\varphi \notin \Gamma $$
4. $$\varphi \wedge \psi \in \Gamma $$ iff $$\varphi \in \Gamma $$ and $$\psi \in \Gamma $$

#### Lemma 6

(Lindenbaum’s Lemma) If $\Gamma \subseteq \mathcal {L}$ is consistent there is a maximally consistent set $\Delta$ such that $\Gamma \subseteq \Delta$.

Let $X_{\textsf{L} }$ denote the set of maximally consistent sets. Define a relation *R* by

$$\begin{aligned} \Gamma R \Delta \iff \forall \varphi \in \mathcal {L}: \textsf{A} \varphi \in \Gamma \implies \varphi \in \Delta \end{aligned}$$

The $(\text{ T}_{\textsf{A} })$ and $(\text{5 }_{\textsf{A} })$ axioms for $\textsf{A}$ show that *R* is an equivalence relation; this is part of the standard proof that S5 is complete for equivalence relations, and we leave the proof to the appendix.

#### Lemma 7

*R* is an equivalence relation.

For $\varphi \in \mathcal {L}$, let $|\varphi | = \{\Gamma \in X_{\textsf{L} } \mid \varphi \in \Gamma \}$ be the *proof set* of $\varphi$. For $\Sigma \in X_{\textsf{L} }$, let $X_\Sigma$ be the equivalence class of $\Sigma$ in *R*, and write $|\varphi |_\Sigma = |\varphi | \cap X_\Sigma$. Using what is essentially the standard proof of the truth lemma for the modal logic **K** with respect to relational semantics, $(\text{ K}_{\textsf{A} })$ yields the following.

#### Lemma 8

Let $$\Sigma \in X_{\textsf{L} }$$. Then

1. For any $\varphi \in \mathcal {L}$, $\textsf{A} \varphi \in \Sigma$ iff $|\varphi |_\Sigma = X_\Sigma$
2. For any $\varphi , \psi \in \mathcal {L}$, $\textsf{A} (\varphi \rightarrow \psi ) \in \Sigma$ iff $|\varphi |_\Sigma \subseteq |\psi |_\Sigma$
3. For any $\varphi , \psi \in \mathcal {L}$, $\textsf{A} (\varphi \leftrightarrow \psi ) \in \Sigma$ iff $|\varphi |_\Sigma = |\psi |_\Sigma$

#### Proof

For brevity we show (1) only, deferring the rest to the appendix.

For the left-to-right direction, suppose $\textsf{A} \varphi \in \Sigma$. Let $\Gamma \in X_\Sigma$. Then $\Sigma R \Gamma$, so clearly $\varphi \in \Gamma$. Hence $|\varphi |_\Sigma = X_\Sigma$.

For the other direction we show the contrapositive. Suppose $\textsf{A} \varphi \notin \Sigma$. Set

$$\begin{aligned} \Gamma _0 = \{\psi \mid \textsf{A} \psi \in \Gamma \} \cup \{\lnot \varphi \}. \end{aligned}$$

We claim $\Gamma _0$ is consistent. If not, without loss of generality there are $\psi _0, \ldots , \psi _n \in \Gamma _0$ such that $\textsf{A} \psi _i \in \Sigma$ for each *i*, and $\vdash \psi _0 \wedge \cdots \wedge \psi _n \rightarrow \varphi .$ By propositional logic, we get $\vdash \psi _0 \rightarrow \cdots \rightarrow \psi _n \rightarrow \varphi$ (where the implication arrows associate to the right) and by $(\text{ Nec}_{\textsf{A} })$, $\vdash \textsf{A} (\psi _0 \rightarrow \cdots \rightarrow \psi _n \rightarrow \varphi ).$ Since $(\text{ K}_{\textsf{A} })$ together with $(\text{ MP})$ says that $\textsf{A}$ distributes over implications, repeated applications gives $\vdash \textsf{A} \psi _0 \rightarrow \cdots \rightarrow \textsf{A} \psi _n \rightarrow \textsf{A} \varphi$ and propositional logic again gives $\vdash \textsf{A} \psi _0 \wedge \cdots \wedge \textsf{A} \psi _n \rightarrow \textsf{A} \varphi .$ But recall that $\textsf{A} \psi _i \in \Sigma$. Hence $\Sigma \vdash \textsf{A} \varphi$. Since $\Sigma$ is maximally consistent, this means $\textsf{A} \varphi \in \Sigma$: contradiction.

So $\Gamma _0$ is consistent. By Lindenbaum’s lemma (Lemma 6), there is a maximally consistent set $\Gamma \supseteq \Gamma _0$. Clearly $\Sigma R \Gamma$, since if $\textsf{A} \psi \in \Sigma$ then $\psi \in \Gamma _0 \subseteq \Gamma$. Moreover, $\lnot \varphi \in \Gamma _0 \subseteq \Gamma$, so by consistency $\varphi \notin \Gamma$. Hence $\Gamma \in X_\Sigma {\setminus } |\varphi |_\Sigma$, and we are done. $\square$

#### Corollary 1

Let $\Sigma \in X_{\textsf{L} }$. For $\Gamma , \Delta \in X_\Sigma$ and $\varphi \in \mathcal {L}$, $\textsf{A} \varphi \in \Gamma$ iff $\textsf{A} \varphi \in \Delta$ and $\textsf{E} \varphi \in \Gamma$ iff $\textsf{E} \varphi \in \Delta$.

We are ready to define the “canonical” model (for each $\Sigma$). Set $\widehat{X}_\Sigma = X_\Sigma \times \mathbb {R}$. This is the step described informally above: we enlargen $X_\Sigma$ by considering uncountably many copies of each point (any uncountable set would do in place of $\mathbb {R}$). The valuation is straightforward: set $\widehat{V}_\Sigma (p) = |p|_\Sigma \times \mathbb {R}$. For the expertise component of the model, say $A \subseteq \widehat{X}_\Sigma$ is *S-closed* iff for all $\varphi \in \mathcal {L}$:

$$\begin{aligned} |\varphi |_\Sigma \times \mathbb {R}\subseteq A \implies |\textsf{S} \varphi |_\Sigma \times \mathbb {R}\subseteq A. \end{aligned}$$

Set $$\widehat{P}_\Sigma = \widehat{P}_\Sigma ^0 \cup \widehat{P}_\Sigma ^1$$, where

$$\begin{aligned} \begin{aligned} \widehat{P}_\Sigma ^0&= \{|\varphi |_\Sigma \times \mathbb {R}\mid \textsf{E} \varphi \in \Sigma \}, \\ \widehat{P}_\Sigma ^1&= \{ A \subseteq \widehat{X}_\Sigma \mid A \text { is S-closed and } \forall \varphi \in \mathcal {L}: A \ne |\varphi |_\Sigma \times \mathbb {R}\}. \end{aligned} \end{aligned}$$

We have a version of the truth lemma for the model $\widehat{M}_\Sigma = (\widehat{X}_\Sigma , \widehat{P}_\Sigma , \widehat{V}_\Sigma )$.

#### Lemma 9

For any $(\Gamma , t) \in \widehat{X}_\Sigma$ and $\varphi \in \mathcal {L}$,

$$\begin{aligned} \widehat{M}_\Sigma , (\Gamma , t) \models \varphi \iff \varphi \in \Gamma , \end{aligned}$$

i.e. $$\Vert \varphi \Vert _{\widehat{M}_\Sigma } = |\varphi |_\Sigma \times \mathbb {R}$$.

#### Proof

By induction. The cases for atomic propositions and the propositional connectives are straightforward by the definition of $\widehat{V}_\Sigma$ and properties of maximally consistent sets. The case for the universal modality $\textsf{A}$ is also straightforward by Lemma 8 and Corollary 1. We treat the cases of $\textsf{E}$ and $\textsf{S}$ formulas.

($\textsf{E}$): First suppose $\textsf{E} \varphi \in \Gamma$. By Corollary 1, $\textsf{E} \varphi \in \Sigma$. Hence $|\varphi |_\Sigma \times \mathbb {R}\in \widehat{P}_\Sigma ^0$. By the induction hypothesis, $\Vert \varphi \Vert _{\widehat{M}_\Sigma } \in \widehat{P}_\Sigma ^0$. Hence $\widehat{M}_\Sigma , (\Gamma , t) \models \textsf{E} \varphi$.

Now suppose $\widehat{M}_\Sigma , (\Gamma , t) \models \textsf{E} \varphi$. Then $\Vert \varphi \Vert _{\widehat{M}_\Sigma } \in \widehat{P}_\Sigma$. By the induction hypothesis, $\Vert \varphi \Vert _{\widehat{M}_\Sigma } = |\varphi |_\Sigma \times \mathbb {R}$. Hence $|\varphi |_\Sigma \times \mathbb {R}\in \widehat{P}_\Sigma$. Since $\widehat{P}_\Sigma ^1$ does not contain any sets of this form, we must have $|\varphi |_\Sigma \times \mathbb {R}\in \widehat{P}_\Sigma ^0$. Therefore there is some $\psi$ such that $\textsf{E} \psi \in \Sigma$ and $|\varphi |_\Sigma \times \mathbb {R}= |\psi |_\Sigma \times \mathbb {R}$. It follows that $|\varphi |_\Sigma = |\psi |_\Sigma$, and Lemma 8 then gives $\textsf{A} (\varphi \leftrightarrow \psi ) \in \Sigma$. By Corollary 1, we have $\textsf{E} \psi \in \Gamma$ and $\textsf{A} (\varphi \leftrightarrow \psi ) \in \Gamma$ too. By $(\text{ RE}_\textsf{E} )$ we get $\textsf{E} \varphi \in \Gamma$ as required.

($\textsf{S}$): First suppose $\textsf{S} \varphi \in \Gamma$. Take $A \in \widehat{P}_\Sigma$ such that $\Vert \varphi \Vert _{\widehat{M}_\Sigma } \subseteq A$. By the induction hypothesis, $|\varphi |_\Sigma \times \mathbb {R}\subseteq A$. There are two cases: either $A \in \widehat{P}_\Sigma ^0$ or $A \in \widehat{P}_\Sigma ^1$.

If $A \in \widehat{P}_\Sigma ^0$, there is $\psi$ such that $A = |\psi |_\Sigma \times \mathbb {R}$ and $\textsf{E} \psi \in \Sigma$. Since $|\varphi |_\Sigma \times \mathbb {R}\subseteq A$, we have $|\varphi |_\Sigma \subseteq |\psi |_\Sigma$. By Lemma 8, $\textsf{A} (\varphi \rightarrow \psi ) \in \Sigma$. By Corollary 1 we have $\textsf{E} \psi , \textsf{A} (\varphi \rightarrow \psi ) \in \Gamma$ too. Applying $(\text{ W}_{\textsf{E} })$ gives $\textsf{S} \varphi \wedge \textsf{E} \psi \rightarrow \psi \in \Gamma$; since $\textsf{S} \varphi , \textsf{E} \psi \in \Gamma$ we have $\textsf{S} \varphi \wedge \textsf{E} \psi \in \Gamma$ and thus $\psi \in \Gamma$. This means $(\Gamma , t) \in |\psi |_\Sigma \times \mathbb {R}= A$, as required.

If $A \in \widehat{P}_\Sigma ^1$, *A* is S-closed by definition. Hence $|\textsf{S} \varphi |_\Sigma \times \mathbb {R}\subseteq A$. Since $\textsf{S} \varphi \in \Gamma$ we get $(\Gamma , t) \in A$ as required.

In either case we have $(\Gamma , t) \in A$. This shows $\widehat{M}_\Sigma , (\Gamma , t) \models \textsf{S} \varphi$.

For the other direction we show the contrapositive. Take any $(\Gamma , t) \in \widehat{X}_\Sigma$ and suppose $\textsf{S} \varphi \notin \Gamma$. We show that $\widehat{M}_\Sigma , (\Gamma , t) \not \models \textsf{S} \varphi$, i.e. there is $A \in \widehat{P}_\Sigma$ such that $\Vert \varphi \Vert _{\widehat{M}_\Sigma } \subseteq A$ but $(\Gamma , t) \notin A$.

First, set

$$\begin{aligned} \mathcal {U} = \{ |\psi |_\Sigma \times \mathbb {R}\mid \psi \in \mathcal {L}\text { and } |\psi |_\Sigma \times \mathbb {R}\not \subseteq |\textsf{S} \varphi |_\Sigma \times \mathbb {R}\}. \end{aligned}$$

Since $\mathcal {L}$ is countable, $\mathcal {U}$ is at most countable. Hence we may write $\mathcal {U} = \{U_n\}_{n \in N}$ for some index set $N \subseteq \mathbb {N}$. Since $U_n \not \subseteq |\textsf{S} \varphi |_\Sigma \times \mathbb {R}$, we may choose some $(\Delta _n, t_n) \in U_n {\setminus } (|\textsf{S} \varphi |_\Sigma \times \mathbb {R})$ for each *n*. Now write

$$\begin{aligned} \mathcal {D} = \{(\Delta _n, t_n)\}_{n \in N} \cup \{(\Gamma , t)\}. \end{aligned}$$

Since *N* is at most countable, so is $\mathcal {D}$. Since $\mathbb {R}$ is uncountable, there is some $s \in \mathbb {R}$ such that $(\Gamma , s) \notin \mathcal {D}$. We necessarily have $s \ne t$. We are ready to define *A*: set

$$\begin{aligned} A = (|\textsf{S} \varphi |_\Sigma \times \mathbb {R}) \cup \{(\Gamma , s)\}. \end{aligned}$$

Note that $(\Gamma , t) \notin A$ since $\textsf{S} \varphi \notin \Gamma$ and $s \ne t$.

Next we show $\Vert \varphi \Vert _{\widehat{M}_\Sigma } \subseteq A$. By the induction hypothesis, this is equivalent to $|\varphi |_\Sigma \times \mathbb {R}\subseteq A$. By $(\text{ T}_{\textsf{S} })$ and $(\text{ Nec}_{\textsf{A} })$, we have $\textsf{A} (\varphi \rightarrow \textsf{S} \varphi ) \in \Sigma$, and consequently $|\varphi |_\Sigma \subseteq |\textsf{S} \varphi |_\Sigma$ by Lemma 8. Hence $|\varphi |_\Sigma \times \mathbb {R}\subseteq |\textsf{S} \varphi |_\Sigma \times \mathbb {R}\subseteq A$ as required.

It only remains to show that $A \in \widehat{P}_\Sigma$. We claim that $A \in \widehat{P}_\Sigma ^1$. First, *A* is S-closed. Indeed, suppose $|\psi |_\Sigma \times \mathbb {R}\subseteq A$. We claim that, in fact, $|\psi |_\Sigma \times \mathbb {R}\subseteq |\textsf{S} \varphi |_\Sigma \times \mathbb {R}$. If not, then by definition of $\mathcal {U}$ there is $n \in N$ such that $|\psi |_\Sigma \times \mathbb {R}= U_n$. Hence $U_n \subseteq A$. This means $(\Delta _n, t_n) \in A$. But $(\Delta _n, t_n) \notin |\textsf{S} \varphi |_\Sigma \times \mathbb {R}$, so we must have $(\Delta _n, t_n) = (\Gamma , s)$. But then $(\Gamma , s) \in \mathcal {D}$: contradiction. So we do indeed have $|\psi |_\Sigma \times \mathbb {R}\subseteq |\textsf{S} \varphi |_\Sigma \times \mathbb {R}$, and thus $|\psi |_\Sigma \subseteq |\textsf{S} \varphi |_\Sigma$. By Lemma 8, $\textsf{A} (\psi \rightarrow \textsf{S} \varphi ) \in \Sigma$.

Now, take any $(\Lambda , u) \in |\textsf{S} \psi |_\Sigma \times \mathbb {R}$. Since $\Lambda \in X_\Sigma$, Corollary 1 gives $\textsf{A} (\psi \rightarrow \textsf{S} \varphi ) \in \Lambda$. By $(\text{ W}_{\textsf{S} })$, $\textsf{S} \psi \rightarrow \textsf{S} \textsf{S} \varphi \in \Lambda$. Since $\Lambda \in |\textsf{S} \psi |_\Sigma$, we get $\textsf{S} \textsf{S} \varphi \in \Lambda$. But then $(\text{4 }_{\textsf{S} })$ gives $\textsf{S} \varphi \in \Lambda$. That is, $(\Lambda , u) \in |\textsf{S} \varphi |_\Sigma \times \mathbb {R}\subseteq A$. This shows $|\textsf{S} \psi |_\Sigma \times \mathbb {R}\subseteq A$, so *A* is S-closed.

Finally, we show that for all $\psi \in \mathcal {L}$, $A \ne |\psi |_\Sigma \times \mathbb {R}$. For contradiction, suppose there is $\psi$ with $A = |\psi |_\Sigma \times \mathbb {R}$. Then since $(\Gamma , s) \in A$, we have $\Gamma \in |\psi |_\Sigma$. But then $(\Gamma , t) \in |\psi |_\Sigma \times \mathbb {R}= A$: contradiction.

This completes the proof that $A \in \widehat{P}_\Sigma ^1$. Thus $\widehat{M}_\Sigma , (\Gamma , t) \not \models \textsf{S} \varphi$, and we are done. $\square$

#### Theorem 4

$\textsf{L}$ is strongly complete^9^ with respect to $\mathbb {M}$.

#### Proof

We show the contrapositive. Suppose $\Gamma \not \vdash \varphi$. Then $\Gamma \cup \{\lnot \varphi \}$ is consistent. By Lindenbaum’s Lemma, there is a maximally consistent set $\Sigma \supseteq \Gamma \cup \{\lnot \varphi \}$. Consider the model $\widehat{M}_\Sigma$. For any $\psi \in \Gamma$ we have $\psi \in \Sigma$, so Lemma 9 (with $t = 0$, say) gives $\widehat{M}_\Sigma , (\Sigma , 0) \models \psi$. Also, $\lnot \varphi \in \Gamma \subseteq \Sigma$ gives $\widehat{M}_\Sigma , (\Sigma , 0) \models \lnot \varphi$, so $\widehat{M}_\Sigma , (\Sigma , 0) \not \models \varphi$. This shows that $\Gamma \not \models \varphi$, and we are done. $\square$

### Extensions of the base logic

We now extend $\textsf{L}$ to obtain axiomatisations of sub-classes of $\mathbb {M}$ corresponding to closure conditions.

To start, consider closure under intersections. It was shown in Proposition 2 that the formula $\textsf{A} (\textsf{S} \varphi \rightarrow \varphi ) \rightarrow \textsf{E} \varphi$ characterises frames closed under intersections. It is perhaps no surprise that adding this as an axiom results in a sound and complete axiomatisation of $\mathbb {M}_{\textsf{int} }$. Formally, let $\textsf{L} _{\textsf{int} }$ be the extension of $\textsf{L}$ with the following axiom

$$\begin{aligned} \textsf{A} (\textsf{S} \varphi \rightarrow \varphi ) \rightarrow \textsf{E} \varphi \quad (\text{ Red}_{\textsf{E} }), \end{aligned}$$

so-named since together with $\textsf{E} \varphi \rightarrow \textsf{A} (\textsf{S} \varphi \rightarrow \varphi )$—which is derivable in $\textsf{L}$—it allows expertise to be reduced to soundness. That is, expertise on $\varphi$ is equivalent to the statement that, in all situations, $\varphi$ is only true up to lack of expertise if it is in fact true.

#### Theorem 5

$\textsf{L} _{\textsf{int} }$ is sound and strongly complete with respect to $\mathbb {M}_{\textsf{int} }$.

#### Proof (sketch)

For soundness, we only need to check that $(\text{ Red}_{\textsf{E} })$ is sound for $\mathbb {M}_{\textsf{int} }$. But this follows from Proposition 2 (1).

For completeness, we take a similar, but simplified, approach to the general case. For brevity we sketch the argument here, with full details deferred to the appendix.

Let $X_{\textsf{L} _{\textsf{int} }}$ be the set of maximally $\textsf{L} _{\textsf{int} }$-consistent sets, and let *R* be defined as before (but now on $X_{\textsf{L} _{\textsf{int} }}$).

Overriding earlier terminology, for any fixed $\Sigma \in X_{\textsf{L} _{\textsf{int} }}$ say $A \subseteq X_\Sigma$ is *S-closed* iff $|\varphi |_\Sigma \subseteq A$ implies $|\textsf{S} \varphi |_\Sigma \subseteq A$ for all $\varphi \in \mathcal {L}$.

The construction of the canonical model for a given $\Sigma$ is now straightforward: set $M_\Sigma = (X_\Sigma , P_\Sigma , V_\Sigma )$, where $A \in P_\Sigma$ iff *A* is S-closed, and $V_\Sigma (p) = |p|_\Sigma$.

It is straightforward to check that $M_\Sigma$ is in $\mathbb {M}_{\textsf{int} }$, i.e. intersections of S-closed sets are S-closed. We also have the truth lemma for $M_\Sigma$: $M_\Sigma , \Gamma \models \varphi \iff \varphi \in \Gamma$.

As usual, the only interesting cases are $\textsf{S}$ and $\textsf{E}$ formulas. For $\textsf{S} \varphi$, the “if” direction is almost immediate by the definition of S-closed and the induction hypothesis. The “only if” direction is shown by contraposition. We show $|\textsf{S} \varphi |_\Sigma$ is S-closed using the analogue of Lemma 8, $(\text{ W}_{\textsf{S} })$ and $(\text{4 }_{\textsf{S} })$; if $\textsf{S} \varphi \notin \Gamma$, $M_\Sigma , \Gamma \not \models \textsf{S} \varphi$ follows from the induction hypothesis and $(\text{ T}_{\textsf{S} })$.

For the “if” direction for $\textsf{E} \varphi$, we use Lemma 8, Corollary 1 and $(\text{ W}_{\textsf{E} })$ to show that $|\varphi |_\Sigma$ is S-closed. For the “only if” direction, the induction hypothesis gives that $|\varphi |_\Sigma$ is S-closed. Since $|\varphi |_\Sigma \subseteq |\varphi |_\Sigma$, we get $|\textsf{S} \varphi |_\Sigma \subseteq |\varphi |_\Sigma$. By Lemma 8 and Corollary 1 again, $\textsf{A} (\textsf{S} \varphi \rightarrow \varphi ) \in \Gamma$, and $\textsf{E} \varphi \in \Gamma$ by $(\text{ Red}_{\textsf{E} })$.

Strong completeness follows using Lindenbaum’s lemma as before. $\square$

Now we add finite unions to the mix. It was shown in Proposition 3 that within class $\mathbb {M}_{\textsf{int} }$, the **K** axiom for the dual operator $\hat{\textsf{S} }\varphi = \lnot \textsf{S} \lnot \varphi$ characterises closure under finite unions. Note that any frame (*X*, *P*) closed under intersections and finite unions is a topological space,^10^ where *P* is the set of *closed* sets. Write $\mathbb {M}_{\textsf{top} }= \mathbb {M}_{\textsf{int} }\cap \mathbb {M}_{\mathsf {finite-unions} }$ for the class of models over such frames. We obtain an axiomatisation of $\mathbb {M}_{\textsf{top} }$ by adding **K** for $\hat{\textsf{S} }$ and a bridge axiom linking $\hat{\textsf{S} }$ and $\textsf{A}$:

$$\begin{aligned} \begin{array}{lr} \hat{\textsf{S} }(\varphi \rightarrow \psi ) \rightarrow (\hat{\textsf{S} }\varphi \rightarrow \hat{\textsf{S} }\psi ) &{} (\text{ K}_{\textsf{S} })\\ \textsf{A} \varphi \rightarrow \hat{\textsf{S} }\varphi &{} (\text{ Inc})\end{array} \end{aligned}$$

Let $\textsf{L} _{\textsf{top} }$ be the extension of $\textsf{L} _{\textsf{int} }$ by $(\text{ K}_{\textsf{S} })$ and $(\text{ Inc})$. Note that $\textsf{L} _{\textsf{top} }$ contains the **KT4** axioms for $\hat{\textsf{S} }$ (recalling that $(\text{ T}_{\textsf{S} })$ and $(\text{4 }_{\textsf{S} })$ are the “diamond” versions of **T** and **4**). Since **KT4** together with the bridge axiom $(\text{ Inc})$ is complete for the class of relational models $\mathbb {M}^*_{\textsf{S4} }$, we can exploit Theorem 3 to obtain completeness of $\textsf{L} _{\textsf{top} }$ with respect to $\mathbb {M}_{\textsf{int} }\cap \mathbb {M}_{\textsf{unions} }$. Since this class is included in $\mathbb {M}_{\textsf{top} }$, we also get completeness with respect to $\mathbb {M}_{\textsf{top} }$.^11^

#### Theorem 6

$\textsf{L} _{\textsf{top} }$ is sound and strongly complete with respect to $\mathbb {M}_{\textsf{top} }$.

#### Proof (sketch)

Soundness of $(\text{ K}_{\textsf{S} })$ for $\mathbb {M}_{\textsf{top} }$ follows from Proposition 3. For $(\text{ Inc})$, suppose $M = (X, P, V) \in \mathbb {M}_{\textsf{top} }$, $x \in X$ and $M, x \models \textsf{A} \varphi$. Then $\Vert \varphi \Vert _M = X$, so $\Vert \lnot \varphi \Vert _M = \emptyset$. By the convention that the empty set is the empty union $\bigcup \emptyset$ (which is a finite union), we have $\emptyset \in P$. Taking $A = \emptyset$ in the definition of the semantics for $\textsf{S}$, we have $\Vert \lnot \varphi \Vert _M \subseteq A$ but clearly $x \notin A$. Hence $M, x \not \models \textsf{S} \lnot \varphi$, so $M, x \models \hat{\textsf{S} }\varphi$.

For completeness we offer only a sketch of the proof, leaving the details to the appendix. First, define a translation $u: \mathcal {L}_{\textsf{K} \textsf{A} }\rightarrow \mathcal {L}$ by sending $\textsf{K} \varphi$ to $\lnot \textsf{S} \lnot u(\varphi )$ and $\textsf{A} \varphi$ to $\textsf{A} u(\varphi )$. Then *u* is the inverse of $t: \mathcal {L}\rightarrow \mathcal {L}_{\textsf{K} \textsf{A} }$ from Sect. 4 up to $\textsf{L} _{\textsf{top} }$-equivalence, in the sense that $\vdash _{\textsf{L} _{\textsf{top} }} \varphi \leftrightarrow u(t(\varphi ))$ for all $\varphi \in \mathcal {L}$. Now let $\textsf{L} _{\textsf{S4A} }$ be the logic over $\mathcal {L}_{\textsf{K} \textsf{A} }$ consisting of **KT4** for $\textsf{K}$, **KT5** for $\textsf{A}$, and a bridge axiom $\textsf{A} \varphi \rightarrow \textsf{K} \varphi$. Then $\textsf{L} _{\textsf{S4A} }$ is strongly complete with respect to $\mathbb {M}^*_{\textsf{S4} }$ (Blackburn et al., 2002, Theorem 7.2). Since the logic $\textsf{L} _{\textsf{top} }$ contains **KT4** for $\hat{\textsf{S} }$ and the bridge axiom, one can show by induction on $\textsf{L} _{\textsf{S4A} }$ proofs that $\vdash _{\textsf{L} _{\textsf{S4A} }}\psi$ implies $\vdash _{\textsf{L} _{\textsf{top} }}u(\psi )$, for all $\psi \in \mathcal {L}_{\textsf{K} \textsf{A} }$. Using the connection between *u* and *t*, we get that $\vdash _{\textsf{L} _{\textsf{S4A} }}t(\varphi )$ implies $\vdash _{\textsf{L} _{\textsf{top} }}\varphi$. Finally, to show strong completeness suppose $\Gamma \models _{\mathbb {M}_{\textsf{top} }} \varphi$. Then by Theorem 3, and using the fact that $\mathbb {M}_{\textsf{top} }\supseteq \mathbb {M}_{\textsf{int} }\cap \mathbb {M}_{\textsf{unions} }$, we have $t(\Gamma ) \models _{\mathbb {M}^*_{\textsf{S4} }} t(\varphi )$; strong completeness gives $t(\Gamma ) \vdash _{\textsf{L} _{\textsf{S4A} }} t(\varphi )$ and thus $\Gamma \vdash _{\textsf{L} _{\textsf{top} }} \varphi$. $\square$

Just as the connection between S4 and $\mathbb {M}_{\textsf{int} }\cap \mathbb {M}_{\textsf{unions} }$ allowed us to obtain a complete axiomatisation of $\mathbb {M}_{\textsf{top} }$, we can axiomatise $\mathbb {M}_{\textsf{int} }\cap \mathbb {M}_{\textsf{compl} }$ by considering S5. Let $\textsf{L} _{\mathsf {int-compl} }$ be the extension of $\textsf{L} _{\textsf{top} }$ with the **5** axiom for $\hat{\textsf{S} }$, which we present in the “diamond” form:

$$\begin{aligned} \textsf{S} \lnot \textsf{S} \varphi \rightarrow \lnot \textsf{S} \varphi \quad (\text{5 }_{\textsf{S} })\end{aligned}$$

#### Theorem 7

$\textsf{L} _{\mathsf {int-compl} }$ is sound and strongly complete with respect to $\mathbb {M}_{\textsf{int} }\cap \mathbb {M}_{\textsf{compl} }$.

The proof of Theorem 7 is similar to that of Theorem 6, and can be found in the appendix.

## The multi-source case

So far we have been able to model the expertise of only a single source. In this section we generalise the setting to handle *multiple* sources. This allows us to consider not only the expertise of different sources individually, but also notions of *collective expertise*. For example, how may sources *combine* their expertise? Is there a suitable notion of *common expertise*? To answer these questions we take inspiration from the well-studied notions of *distributed knowledge* and *common knowledge* from epistemic logic (Fagin et al., 2003), and establish connections between collective expertise and collective knowledge.

### Collective knowledge

Let $\mathcal {J}$ be a finite, non-empty set of sources. Turning briefly to epistemic logic interpreted under relational semantics, we recount several notions of collective knowledge. First, a *multi-source relational model* is a triple $M^* = (X, \{R_j\}_{j \in \mathcal {J}}, V)$, where $R_j$ is a binary relation on *X* for each *j*. Consider the following knowledge operators (Fagin et al., 2003):

- $\textsf{K} _j\varphi$ (individual knowledge): for $j \in J$ and a formula $\varphi$, set
  $$\begin{aligned} M^*, x \models \textsf{K} _j\varphi \iff \forall y \in X: x{R_j}y \implies M^*, y \models \varphi . \end{aligned}$$
  This is the straightforward adaptation of knowledge in the single-source case to the multi-source setting.
- $\textsf{K} ^{\textsf{dist} }_J\varphi$ (distributed knowledge): for $J \subseteq \mathcal {J}$ non-empty, set
  $$\begin{aligned} M^*, x \models \textsf{K} ^{\textsf{dist} }_J\varphi \iff \forall y \in X: (x, y) \in \bigcap _{j \in J}{R_j} \implies M^*, y \models \varphi . \end{aligned}$$
  That is, knowledge of $\varphi$ is distributed among the sources in *J* if, by combining their accessibility relations $R_j$, all states possible at *x* satisfy $\varphi$. Here the $R_j$ are combined by taking their intersection: a state *y* is possible according to the group at *x* iff *every* source in *J* considers *y* possible at *x*.
- $\textsf{K} ^{\textsf{sh} }_J\varphi$ (shared knowledge)^12^: for $J \subseteq \mathcal {J}$ non-empty, set
  $$\begin{aligned} M^*, x \models \textsf{K} ^{\textsf{sh} }_J\varphi \iff \forall j \in J: M^*, x \models \textsf{K} _j\varphi . \end{aligned}$$
  That is, a group *J* have shared knowledge of $\varphi$ exactly when each agent in *J* knows $\varphi$. Thus we have $\textsf{K} ^{\textsf{sh} }_J\varphi \equiv \bigwedge _{j \in J}\textsf{K} _j\varphi$.
- $\textsf{K} ^{\textsf{com} }_J\varphi$ (common knowledge): write $\textsf{K} _J^1\varphi$ for $\textsf{K} ^{\textsf{sh} }_J\varphi$, and for $n \in \mathbb {N}$ write $\textsf{K} _J^{n + 1}\varphi$ for $\textsf{K} ^{\textsf{sh} }_J\textsf{K} _J^n\varphi$. Then
  $$\begin{aligned} M^*, x \models \textsf{K} ^{\textsf{com} }_J\varphi \iff \forall n \in \mathbb {N}: M^*, x \models \textsf{K} _J^n\varphi . \end{aligned}$$
  Here $\textsf{K} _J^1\varphi$ says that everyone in *J* knows $\varphi$, $\textsf{K} _J^2\varphi$ says that everybody in *J* knows that everybody in *J* knows $\varphi$, and so on. There is common knowledge of $\varphi$ among *J* if this nesting of “everybody knows” holds for any order *n*.

In what follows we write $\mathcal {L}_{\textsf{K} \textsf{A} }^{\mathcal {J}}$ for the language formed from $\textsf{Prop}$ with knowledge operators $\textsf{K} _j$, $\textsf{K} ^{\textsf{dist} }_J$, $\textsf{K} ^{\textsf{sh} }_J$ and $\textsf{K} ^{\textsf{com} }_J$, for $j \in \mathcal {J}$ and $J \subseteq \mathcal {J}$ non-empty, and the universal modality $\textsf{A}$.

### Collective expertise

Returning to expertise semantics, define a *multi-source expertise model* as a triple $M = (X, \{P_j\}_{j \in \mathcal {J}}, V)$, where $P_j \subseteq 2^X$ is the collection of expertise sets for source *j*. Say *M* is closed under intersections, unions, complements etc. if each $P_j$ is. Since the connection between expertise and S4 knowledge (Theorem 3) holds for expertise models closed under unions and intersections, we restrict attention to this class of (multi-source) models in this section.

The counterpart of individual knowledge—individual expertise—is straightforward: we may simply introduce expertise and soundness operators $\textsf{E} _j$ and $\textsf{S} _j$ for each source $j \in \mathcal {J}$, and interpret $\textsf{E} _j\varphi$ and $\textsf{S} _j\varphi$ as in the single-source case using $P_j$. For notions of collective expertise and soundness, we define new collections $P_J$ by combining the $P_j$ in an appropriate way.

#### Distributed expertise

For distributed expertise, the intuition is clear: the sources in a group *J* should combine their expertise collections $P_j$ to form a larger collection $P^{\textsf{dist} }_J$. A first candidate for $P^{\textsf{dist} }_J$ would therefore be $\bigcup _{j \in J}{P_j}$. However, since we assume each $P_j$ is closed under unions and intersections, we suppose that each source *j* has the cognitive or computational capacity to combine expertise sets $A \in P_j$ by taking unions or intersections. We argue that the same should be possible for the group *J* as a whole, and therefore let $P^{\textsf{dist} }_J$ be the closure of $\bigcup _{j \in J}{P_j}$ under unions and intersections:

$$\begin{aligned} P^{\textsf{dist} }_J = \bigcap \left\{ P' \supseteq \bigcup _{j \in J}{P_j} \mid P' \text { is closed under unions and intersections} \right\} . \end{aligned}$$

Note that $P^{\textsf{dist} }_J$ is closed under unions and intersections, and $P_j \subseteq P^{\textsf{dist} }_J$ for all $j \in J$ (in fact, $P^{\textsf{dist} }_J$ is the smallest set with these properties). While $P^{\textsf{dist} }_J$ depends on the model *M*, we suppress this from the notation.

$P^{\textsf{dist} }_J$ also has a topological interpretation. As in Sect. 4, each $P_j$ gives rise to an Alexandrov topology $\tau _j$ (where $P_j$ are the closed sets) if it is closed under unions and intersections. By the aforementioned properties, $\tau ^\textsf{dist} _J$ corresponds to the coarsest Alexandrov topology finer than each $\tau _j$. On the other hand, since the join (in the lattice of topologies on *X*) of finitely many Alexandrov topologies is again Alexandrov (Steiner, 1966, Theorems 2.4, 2.5), it follows that $\tau ^\textsf{dist} _J$ is equal to the join $\bigvee _{j \in J}{\tau _j}$.^13^

Now, recall from Theorem 3 that our semantics for expertise and soundness is connected to relational semantics via the mapping $P \mapsto R_P$ (Definition 3). The following result shows that $P^{\textsf{dist} }_J$ corresponds to distributed knowledge under this mapping. For ease of notation, write $R^{\textsf{dist} }_J$ for $R_{P^{\textsf{dist} }_J}$ and $R_j$ for $R_{P_j}$.

##### Proposition 4

For any multi-source expertise model *M* and $J \subseteq \mathcal {J}$ non-empty,

$$\begin{aligned} R^{\textsf{dist} }_J = \bigcap _{j \in J}{R_j}. \end{aligned}$$

##### Proof

“$\subseteq$”: Suppose $x{R^{\textsf{dist} }_J}y$. Let $j \in J$. We need to show $x{R_j}y$. Take any $A \in P_j$ such that $y \in A$. Then $A \in P^{\textsf{dist} }_J$, so $x{R^{\textsf{dist} }_J}y$ gives $x \in A$. Hence $x{R_j}y$.

“$\supseteq$”: Suppose $(x, y) \in \bigcap _{j \in J}{R_j}$, i.e. $x{R_j}y$ for all $j \in J$. Set

$$\begin{aligned} P' = \{A \in P^{\textsf{dist} }_J \mid y \in A \implies x \in A\} \subseteq P^{\textsf{dist} }_J. \end{aligned}$$

Then $P' \supseteq \bigcup _{j \in J}{P_j}$, since if $j \in J$ and $A \in P_j$ then $A \in P^{\textsf{dist} }_J$ and $y \in A$ implies $x \in A$ by $x{R_j}y$. We claim $P'$ is closed under intersections. Suppose $\{A_i\}_{i \in I} \subseteq P'$ and write $A = \bigcap _{i \in I}{A_i}$. Since $P' \subseteq P^{\textsf{dist} }_J$ and $P^{\textsf{dist} }_J$ is closed under intersections, $A \in P^{\textsf{dist} }_J$. Suppose $y \in A$. Then $y \in A_i$ for each *i*, so $x \in A_i$ by the defining property of $P'$. Hence $x \in \bigcap _{i \in I}{A_i} = A$. This shows $A \in P'$ as desired. A similar argument shows that $P'$ is also closed under unions.

We see from the definition of $P^{\textsf{dist} }_J$ that $P^{\textsf{dist} }_J \subseteq P'$, so in fact $P' = P^{\textsf{dist} }_J$. It now follows that $x{R^{\textsf{dist} }_J}y$: for any $A \in P^{\textsf{dist} }_J$ with $y \in A$ we have $A \in P'$, so $x \in A$ also. $\square$

#### Common expertise

Common expertise admits a straightforward definition: simply take the expertise sets in common with all $P_j$:

$$\begin{aligned} P^{\textsf{com} }_J = \bigcap _{j \in J}{P_j}. \end{aligned}$$

If each $P_j$ is closed under unions and intersections, then so too is $P^{\textsf{com} }_J$.

At first this may appear *too* straightforward. The form of the definition is closer to *shared* knowledge than to common knowledge. But in fact, shared knowledge has *no* expertise counterpart which admits the type of connection established in Theorem 3. Indeed, shared knowledge may fail positive introspection (axiom **4**: $\textsf{K} \varphi \rightarrow \textsf{K} \textsf{K} \varphi$), but we have seen that the knowledge derived from expertise and soundness satisfies S4 (when the collection of expertise sets is closed under unions and complements).

However, this problem is only apparent in the translation of $\textsf{S} \varphi$ as $\lnot \textsf{K} \lnot \varphi$. For our translation of $\textsf{E} \varphi$ as $\textsf{A} (\lnot \varphi \rightarrow \textsf{K} \lnot \varphi )$, the universal quantification via $\textsf{A}$ dissolves the differences between shared and common knowledge.

##### Proposition 5

Let $\varphi \in \mathcal {L}_{\textsf{K} \textsf{A} }^{\mathcal {J}}$ and let $J \subseteq \mathcal {J}$ be non-empty. Then

$$\begin{aligned} \textsf{A} (\lnot \varphi \rightarrow \textsf{K} ^{\textsf{com} }_J\lnot \varphi ) \equiv \textsf{A} (\lnot \varphi \rightarrow \textsf{K} ^{\textsf{sh} }_J\lnot \varphi ). \end{aligned}$$

We leave the proof to the appendix. Proposition 5 shows that when interpreting collective expertise on $\varphi$ as collective refutation of $\varphi$ whenever $\varphi$ is false, there is no difference between using common knowledge and just shared knowledge.

We now confirm that $P^{\textsf{com} }_J$ does indeed correspond to common knowledge. First we recall a well-known result from Fagin et al. (2003). In what follows, write $R^+= \bigcup _{n \in \mathbb {N}}{R^n}$ for the transitive closure of *R*.

##### Lemma 10

(Fagin et al., 2003, Lemma 2.2.1) Let $M^* = (X, \{R_j\}_{j \in \mathcal {J}}, V)$ be a multi-source relational model and $J \subseteq \mathcal {J}$ non-empty. Write $R' = \left( \bigcup _{j \in J}{R_j}\right) ^+$. Then for all $x \in X$ and $\varphi \in \mathcal {L}_{\textsf{K} \textsf{A} }^{\mathcal {J}}$:

$$\begin{aligned} M^*, x \models \textsf{K} ^{\textsf{com} }_J\varphi \iff \forall y \in X: x{R'}y \implies M^*, y \models \varphi . \end{aligned}$$

By Lemma 10, common knowledge has an interpretation in terms of the usual relational semantics for knowledge, where we use the transitive closure of the union of the accessibility relations of the sources in *J*. Writing $R^{\textsf{com} }_J$ for $R_{P^{\textsf{com} }_J}$, we have the following.

##### Proposition 6

Let *M* be a multi-source model closed under unions and intersections. Then for $J \subseteq \mathcal {J}$ non-empty, $R^{\textsf{com} }_J = \left( \bigcup \nolimits _{j \in J}{R_j} \right) ^{+}.$

##### Proof

Write $R' = (\bigcup _{j \in J}{R_j})^{+}$. Note that $R^{\textsf{com} }_J$ is reflexive and transitive by Lemma 3 (1). $R'$ is transitive by its definition as a transitive closure, and reflexive since each $R_j$ is (and $J \ne \emptyset$).

It is therefore sufficient by Lemma 2 to show that any set is downwards closed wrt $R^{\textsf{com} }_J$ iff it is downwards closed wrt $R'$. Since each $P_j$ is closed under unions and intersections, so too is $P^{\textsf{com} }_J$. Using Lemma 3 (2), we have

$$\begin{aligned} \begin{aligned} A \text { downwards closed wrt } R^{\textsf{com} }_J&\iff A \in P^{\textsf{com} }_J \\&\iff \forall j \in J: A \in P_j \\&\iff \forall j \in J: A \text { downwards closed wrt } R_j \\&\iff A \text { downwards closed wrt } \bigcup \nolimits _{j \in J}{R_j} \\&\iff A \text { downwards closed wrt } R' \end{aligned} \end{aligned}$$

where the last step uses the fact that *A* is downwards closed with respect to some relation if and only if it is downwards closed with respect to the transitive closure. $\square$

#### Collective semantics

We now formally define the syntax and semantics of collective expertise. Let $\mathcal {L}^{\mathcal {J}}$ be the language defined by the following grammar:

$$\begin{aligned} \varphi {:}{:}{=} p \mid \varphi \wedge \varphi \mid \lnot \varphi \mid \textsf{E} _j\varphi \mid \textsf{S} _j\varphi \mid \textsf{E} _J^g\varphi \mid \textsf{S} _J^g\varphi \mid \textsf{A} \varphi \end{aligned}$$

for $p \in \textsf{Prop}$, $j \in \mathcal {J}$, $g \in \{\textsf{dist} , \textsf{com} \}$ and $J \subseteq \mathcal {J}$ non-empty. For a multi-source expertise model $M = (X, \{P_j\}_{j \in \mathcal {J}}, V)$, define the satisfaction relation as before for atomic propositions, propositional connectives and $\textsf{A}$, and set

$$\begin{aligned} \begin{array}{lllr} M, x &{}\models E_j\varphi &{}\iff \Vert \varphi \Vert _M \in P_j \\ M, x &{}\models E_J^g\varphi &{}\iff \Vert \varphi \Vert _M \in P_J^g &{} (g \in \{\textsf{dist} , \textsf{com} \}) \\ M, x &{}\models S_j\varphi &{}\iff \forall A \in P_J: \Vert \varphi \Vert _M \subseteq A \implies x \in A \\ M, x &{}\models S_J^g\varphi &{}\iff \forall A \in P_J^g: \Vert \varphi \Vert _M \subseteq A \implies x \in A &{} (g \in \{\textsf{dist} , \textsf{com} \}) \end{array} \end{aligned}$$

Note that expertise and soundness are interpreted as before, but with respect to different collections *P*. Consequently, the interactions shown in Proposition 1 still hold for individual and collective notions of expertise and soundness.

##### Example 3

Extending Examples 1 and 2, consider $\mathcal {J}= \{\textsf{econ} , \textsf{dr} , \textsf{analyst} \}$, where $\textsf{econ}$ is the economist, $\textsf{dr}$ is a doctor with expertise on *i* only, and $\textsf{analyst}$ has access to aggregate data distinguishing three levels of virus activity: minimal ($\lnot i \wedge \lnot d$), high ($(i \vee d) \wedge \lnot (i \wedge d)$) and very high ($i \wedge d$). This can be modelled by a multi-source model *M* with *X*, *V* and $P_\textsf{econ}$ as in Example 2, and $P_\textsf{dr} = \{\emptyset , X, \{ipd, ip, id, i\}, \{pd, p, d, \emptyset \}\}$, $P_\textsf{analyst}$ is the closure under unions of $\{\emptyset , X, \{ipd, id\}, \{ip, pd, i, d\}, \{p, \emptyset \}\}$.

Note that neither $\textsf{dr}$ nor $\textsf{analyst}$ have expertise on *d* individually. However, if $\textsf{dr}$ can communicate whether or not *i* holds, this gives $\textsf{analyst}$ enough information to disambiguate the “high activity” case and therefore determine *d*. Indeed, we have $\Vert d\Vert = \Vert i \wedge d\Vert \cup (\Vert i \vee d\Vert {\setminus } \Vert i \wedge d\Vert \cap \Vert \lnot i\Vert )$, which is formed by unions and intersections from $P_\textsf{dr} \cup P_\textsf{analyst}$, and thus $\Vert d\Vert \in P^{\textsf{dist} }_{\{\textsf{dr} , \textsf{analyst} \}}$. Hence $M \models \textsf{E} _{\{\textsf{dr} , \textsf{analyst} \}}^\textsf{dist} d$.

Similarly, $\textsf{dr}$ and $\textsf{analyst}$ have distributed expertise on $\lnot d$. Bringing back $\textsf{econ}$, the grand coalition $\mathcal {J}$ have distributed expertise on the original report $p \wedge \lnot d$ from Example 1. Consequently, the report is no longer sound at “actual” state *idp*: all sources together have sufficient expertise to know it is false.

The following validities express properties specific to collective expertise.

##### Proposition 7

The following formulas are valid.

1. For $$j \in J$$, $$\textsf{E} _j\varphi \rightarrow \textsf{E} _J^\textsf{dist} \varphi $$
2. $$\textsf{E} _J^\textsf{com} \varphi \leftrightarrow \bigwedge _{j \in J}{\textsf{E} _j\varphi }$$
3. $$\textsf{S} _J^\textsf{com} \varphi \leftrightarrow \bigvee _{j \in J}\textsf{S} _j\textsf{S} _J^\textsf{com} \varphi $$
4. $\textsf{E} _{\{j\}}^\textsf{dist} \varphi \leftrightarrow \textsf{E} _j\varphi$ is valid on $\mathbb {M}_{\textsf{int} }^{\mathcal {J}}\cap \mathbb {M}_{\textsf{unions} }^{\mathcal {J}}$

##### Proof

We prove only (3); the others are straightforward. The right implication is valid since $\psi \rightarrow \textsf{S} _j\psi$ is, with $\psi$ set to $\textsf{S} _J^\textsf{com} \varphi$ and $j \in J$ arbitrary (recall *J* is non-empty).

For the left implication, suppose there is $j \in J$ with $M, x \models \textsf{S} _j\textsf{S} _J^\textsf{com} \varphi$. Then $x \in \bigcap \{A \in P_j \mid \Vert \textsf{S} _J^\textsf{com} \varphi \Vert _M \subseteq A\}$. Now take $B \in P_J^\textsf{com}$ such that $\Vert \varphi \Vert _M \subseteq B$. Note that if $y \in \Vert \textsf{S} _J^\textsf{com} \varphi \Vert$ then $y \in B$ by the definition of the semantics for $\textsf{S} _J^\textsf{com}$, so $\Vert \textsf{S} _J^\textsf{com} \varphi \Vert _M \subseteq B$. Since $B \in P_J^\textsf{com} \subseteq P_j$, we get $x \in B$. This shows $M, x \models \textsf{S} _J^\textsf{com} \varphi$.

Validity (3) comes from the *fixed-point axiom* for common knowledge: $\textsf{K} ^{\textsf{com} }_J\varphi \leftrightarrow \textsf{K} ^{\textsf{sh} }_J(\varphi \wedge \textsf{K} ^{\textsf{com} }_J\varphi )$. Our version says $\textsf{S} _J^\textsf{com} \varphi$ is a fixed-point of the function $\theta \mapsto \bigvee _{j \in J}{\textsf{S} _j\theta }$. In words, $\varphi$ is true up to lack of *common* expertise iff there is some source for whom $\textsf{S} _J^\textsf{com} \varphi$ is true up to their lack of (individual) expertise.

As promised, there is a tight link between our notions of collective expertise and knowledge. Define a translation $t: \mathcal {L}^{\mathcal {J}}\rightarrow \mathcal {L}_{\textsf{K} \textsf{A} }^{\mathcal {J}}$ inductively by

$$\begin{aligned} t(\textsf{E} _j\varphi )&= \textsf{A} (\lnot t(\varphi ) \rightarrow \textsf{K} _j\lnot t(\varphi )) \\ t(\textsf{E} _J^g\varphi )&= \textsf{A} (\lnot t(\varphi ) \rightarrow \textsf{K} _J^g\lnot t(\varphi )) \quad (g \in \{\textsf{dist} , \textsf{com} \}) \\ t(\textsf{S} _j\varphi )&= \lnot \textsf{K} _j\lnot t(\varphi ) \\ t(\textsf{S} _J^g\varphi )&= \lnot \textsf{K} _J^g\lnot t(\varphi ) \quad (g \in \{\textsf{dist} , \textsf{com} \}) \end{aligned}$$

where the other cases are as for *t* in Sect. 4. This is essentially the same translation as before, but with the various types of expertise and soundness matched with their knowledge counterparts. We have an analogue of Theorem 3.

##### Theorem 8

The mapping $f: \mathbb {M}_{\textsf{int} }^{\mathcal {J}}\cap \mathbb {M}_{\textsf{unions} }^{\mathcal {J}}\rightarrow \mathbb {M}^{\mathcal {J}}_{\textsf{S4} }$ given by $(X, \{P_j\}_{j \in \mathcal {J}}, V) \mapsto (X, \{R_{P_j}\}_{j \in \mathcal {J}}, V)$ is bijective, and for $x \in X$ and $\varphi \in \mathcal {L}^{\mathcal {J}}$:

$$\begin{aligned} M, x \models \varphi \iff f(M), x \models t(\varphi ). \end{aligned}$$

Moreover, the restriction of this map to $\mathbb {M}_{\textsf{int} }^{\mathcal {J}}\cap \mathbb {M}_{\textsf{compl} }^{\mathcal {J}}$ is a bijection into $\mathbb {M}^{\mathcal {J}}_{\textsf{S5} }$.

##### Proof

That the map is bijective follows easily from Theorems 1 and 2. For the stated property we proceed by induction on $\mathcal {L}^{\mathcal {J}}$ formulas. As in Theorem 3, the cases for atomic propositions, propositional connectives and $\textsf{A}$ are straightforward. For expertise and soundness, the argument in the proof of Theorem 3 showed that $\textsf{E} \varphi$ and $\textsf{S} \varphi$ interpreted via some collection *P* is equivalent to $t(\textsf{E} \varphi )$ and $t(\textsf{S} \varphi )$ interpreted wrt relational semantics via $R_P$. It is therefore sufficient to show that for each notion of individual and collective expertise interpreted in *M* via *P*, its corresponding notion of individual or collective knowledge (used in the translation *t*) is interpreted in *f*(*M*) via $R_P$. This is self-evident for individual expertise. For distributive expertise this was shown in Proposition 4. For common expertise this was shown in Lemma 10 and Proposition 6.

Theorem 8 can be used to adapt any sound and complete axiomatisation for $\mathbb {M}^{\mathcal {J}}_{\textsf{S4} }$ (resp., $\mathbb {M}^{\mathcal {J}}_{\textsf{S5} }$) over the language $\mathcal {L}_{\textsf{K} \textsf{A} }^{\mathcal {J}}$ to obtain an axiomatisation for $\mathbb {M}_{\textsf{int} }^{\mathcal {J}}\cap \mathbb {M}_{\textsf{unions} }^{\mathcal {J}}$ (resp., $\mathbb {M}_{\textsf{int} }^{\mathcal {J}}\cap \mathbb {M}_{\textsf{compl} }^{\mathcal {J}}$) over $\mathcal {L}^{\mathcal {J}}$, in the same way as we did earlier when adapting S4 and S5 in Theorems 6 and 7.

## Conclusion

This paper presented a simple modal logic framework to reason about the expertise of information sources and soundness of information, generalising the framework of Singleton (2021). We investigated both conceptual and technical issues, establishing several completeness for various classes of expertise models. The connection with epistemic logic showed how expertise and soundness may be given precise interpretations in terms of knowledge; if expertise is closed under intersections and unions this results in S4 knowledge, and closure under complements strengthens this to S5. Finally, we extended the framework to handle multiple sources and studied notions of collective expertise.

There are many directions for future work. First, our approach allows one to reason about soundness of information only if the extent of a source’s expertise is known up-front. In practical situations it is more likely that one has to *estimate* a source’s expertise, e.g. on the basis of previous reports (Dastani et al., 2004; Hunter, 2021). A first attempt in this direction has been proposed in Singleton and Booth (2022).

Expertise is also not static: it may change over time as sources learn and acquire new evidence. To model this one could introduce *dynamic expertise operators*, as in Dynamic Epistemic Logic. One source of inspiration here is *dynamic evidence logics*  (van Benthem et al., 2014; van Benthem & Pacuit, 2011), which study how evidence (and beliefs formed on the basis of evidence) change over time. Such logics also use neighbourhood semantics to interpret evidence modalities, which is technically (and possibly conceptually) similar to our semantics for expertise.

Finally, there is scope to study the interaction between expertise and *trust*, which has been extensively studied from a logical perspective (Booth & Hunter, 2018; Herzig et al., 2010; Liau, 2003; Lorini et al., 2014). Intuitively, source *i* should trust *j* on $\varphi$ if *i* believes that *j* has expertise on $\varphi$. “Belief in expertise” in this manner is not particularly meaningful in the current framework, since $\textsf{E} _j\varphi$ either holds everywhere or nowhere. Future work could extend the semantics to allow the expertise collection $P_j$ to vary between states, so as to model one source’s uncertainty about the expertise of another.

## Footnotes

1 Note that we could instead consider the dual case: expertise means being able to *verify* when a proposition is true.

2 https://en.wikipedia.org/wiki/Rogers_Commission_Report.

3 https://github.com/joesingo/expertise-and-information/blob/master/latex/appendix.pdf.

4 https://github.com/joesingo/expertise-and-information.

5 For this to be the case, *P* must be closed under intersections and finite unions, and contain both the empty set and *X* itself. We will turn to these closure properties in Sect. :3.

6 Our semantics for soundness is therefore dual to the *interior semantics* for modal logic, where $\Box \varphi$ is true at *x* iff *x* lies in the interior of $\Vert \varphi \Vert$.

7 Indeed, consider $M = (X, P, V)$, where $X = \{1, 2, 3\}$, $P = \{\{1, 2\}, \{2, 3\}\}$ and $V(p) = \{1, 2\}$, $V(q) = \{2, 3\}$ for some fixed $p, q \in \textsf{Prop}$. Let $M'$ be as in Lemma 1. Then $M', 1 \models \textsf{E} (p \wedge q)$ and $M, 1 \not \models \textsf{E} (p \wedge q)$, but *M* and $M'$ agree on $\mathcal {L}_{\textsf{S} \textsf{A} }$ formulas. Hence $\textsf{E} (p \wedge q)$ is not equivalent to any $\mathcal {L}_{\textsf{S} \textsf{A} }$ formula.

8 In fact, the interior semantics has an intrinsic epistemic interpretation (without appeal to any link with relational semantics) if one views open sets as *evidence* (Özgün, 2017, p. 24).

9 That is, for all sets $\Gamma \subseteq \mathcal {L}$ and $\varphi \in \mathcal {L}$, if $\Gamma \models \varphi$ then $\Gamma \vdash \varphi$.

10 By the convention that the empty intersection is the whole space *X* and the empty union is $\emptyset$, we have $X, \emptyset \in P$ too.

11 Note that **KT4** is also complete for topological spaces with respect to the interior semantics (van Benthem & Bezhanishvili, 2007).

12 In Fagin et al. (2003), shared knowledge is denoted $\textsf{E}_J\varphi$ for “everybody knows $\varphi$”. We opt to use the term “shared” knowledge to avoid conflict with our notation for expertise.

13 We thank an anonymous reviewer for bringing this to our attention.

## References

[CR1] Blackburn P, De Rijke M, Venema Y (2002). Modal logic.

[CR2] Blum C, Zuber CI (2016). Liquid democracy: Potentials, problems, and perspectives. Journal of Political Philosophy.

[CR3] Booth R, Hunter A (2018). Trust as a precursor to belief revision. JAIR.

[CR4] Chi MT, Glaser R, Farr MJ (2014). The nature of expertise.

[CR5] Collins H, Evans R (2008). Rethinking expertise.

[CR6] Dastani, M., Herzig, A., Hulstijn, J., & van der Torre, L. (2004). Inferring trust. In J. Leite & P. Torroni (Eds.), *CLIMA* (pp. 144–160). Springer.

[CR7] Ericsson KA, Towne TJ (2010). Expertise. WIREs Cognitive Science.

[CR8] Fagin R, Moses Y, Halpern JY, Vardi MY (2003). Reasoning about knowledge.

[CR9] Goldman AI (2018). Expertise. Topoi.

[CR10] Goranko V, Passy S (1992). Using the universal modality: Gains and questions. Journal of Logic and Computation.

[CR11] Herzig A, Lorini E, Hübner JF, Vercouter L (2010). A logic of trust and reputation. Logic Journal of the IGPL.

[CR12] Hunter A, Pham DN, Theeramunkong T, Governatori G, Liu F (2021). Building trust for belief revision. Pricai 2021: Trends in artificial intelligence.

[CR13] Kilov D (2021). The brittleness of expertise and why it matters. Synthese.

[CR14] Liau C-J (2003). Belief, information acquisition, and trust in multi-agent systems—a modal logic formulation. Artificial Intelligence.

[CR15] Llewellyn S (2020). Covid-19: How to be careful with trust and expertise on social media. BMJ.

[CR16] Lorini, E., Jiang, G., & Perrussel, L. (2014). Trust-based belief change. In *Proc. ECAI* (pp. 549–554).

[CR17] Montague R (1970). Universal grammar. Theoria.

[CR18] Özgün, A. (2017). *Evidence in epistemic logic? A topological perspective (Thesis)*. University of Amsterdam, Université de Lorraine and IILC.

[CR19] Pacuit E (2017). Neighborhood semantics for modal logic.

[CR20] Scott, D. (1970). Advice on modal logic. In K. Lambert (Ed.), *Philosophical problems in logic: Some recent developments* (pp. 143–173). Springer. 10.1007/978-94-010-3272-8_7.

[CR21] Singleton, J. (2021). A logic of expertise. ESSLLI 2021 Student Session. Retrieved from https://arxiv.org/abs/2107.10832

[CR22] Singleton, J., & Booth, R. (2022). Who’s the expert? On multi-source belief change. *Proceedings of the 19th international conference on principles of knowledge representation and reasoning* (pp. 331–340). Retrieved from 10.24963/kr.2022/33

[CR23] Steiner AK (1966). The lattice of topologies: Structure and complementation. Transactions of the American Mathematical Society.

[CR24] van Benthem J, Bezhanishvili G, Aiello M, Pratt-Hartmann I, Van Benthem J (2007). Modal logics of space. Handbook of spatial logics.

[CR25] van Benthem J, Pacuit E (2011). Dynamic logics of evidence-based beliefs. Studia Logica.

[CR26] van Benthem J, Fernández-Duque D, Pacuit E (2014). Evidence and plausibility in neighborhood structures. Annals of Pure and Applied Logic.

[CR27] van Dijck J, Alinejad D (2020). Social media and trust in scientific expertise: Debating the covid-19 pandemic in the Netherlands. Social Media + Society.

[CR28] Whyte KP, Crease RP (2010). Trust, expertise, and the philosophy of science. Synthese.

[CR29] Xaudiera, S., & Cardenal, A. S. (2020). Ibuprofen narratives in five European countries during the covid-19 pandemic. *Harvard Kennedy School Misinformation Review*, *1*(3). Retrieved from https://misinforeview.hks.harvard.edu/article/ibuprofen-narratives-infive-european-countries-during-the-covid-19-pandemic/

